# MetMatch: A Semi-Automated Software Tool for the Comparison and Alignment of LC-HRMS Data from Different Metabolomics Experiments

**DOI:** 10.3390/metabo6040039

**Published:** 2016-11-02

**Authors:** Stefan Koch, Christoph Bueschl, Maria Doppler, Alexandra Simader, Jacqueline Meng-Reiterer, Marc Lemmens, Rainer Schuhmacher

**Affiliations:** 1 Center for Analytical Chemistry, Department of Agrobiotechnology (IFA-Tulln), University of Natural Resources and Life Sciences, Vienna (BOKU), Tulln an der Donau 3430, Austria; stefan.koch@boku.ac.at (S.K.); christoph.bueschl@boku.ac.at (C.B.); maria.doppler@boku.ac.at (M.D.); alexandra.simader@boku.ac.at (A.S.); 2 Institute for Biotechnology in Plant Production, Department of Agrobiotechnology (IFA-Tulln), University of Natural Resources and Life Sciences, Vienna (BOKU), Tulln an der Donau 3430, Austria; jacqueline.reiterer@boku.ac.at (J.M.-R.); marc.lemmens@boku.ac.at (M.L.)

**Keywords:** feature detection, retention time correction, metabolite data matching

## Abstract

Due to its unsurpassed sensitivity and selectivity, LC-HRMS is one of the major analytical techniques in metabolomics research. However, limited stability of experimental and instrument parameters may cause shifts and drifts of retention time and mass accuracy or the formation of different ion species, thus complicating conclusive interpretation of the raw data, especially when generated in different analytical batches. Here, a novel software tool for the semi-automated alignment of different measurement sequences is presented. The tool is implemented in the Java programming language, it features an intuitive user interface and its main goal is to facilitate the comparison of data obtained from different metabolomics experiments. Based on a feature list (i.e., processed LC-HRMS chromatograms with mass-to-charge ratio (*m*/*z*) values and retention times) that serves as a reference, the tool recognizes both *m*/*z* and retention time shifts of single or multiple analytical datafiles/batches of interest. MetMatch is also designed to account for differently formed ion species of detected metabolites. Corresponding ions and metabolites are matched and chromatographic peak areas, *m*/*z* values and retention times are combined into a single data matrix. The convenient user interface allows for easy manipulation of processing results and graphical illustration of the raw data as well as the automatically matched ions and metabolites. The software tool is exemplified with LC-HRMS data from untargeted metabolomics experiments investigating phenylalanine-derived metabolites in wheat and T-2 toxin/HT-2 toxin detoxification products in barley.

## 1. Introduction

Technological advances in analytical techniques have helped metabolomics to emerge into a valuable discipline for studying metabolic phenotypes caused by various perturbations such as environmental influences, biotic/abiotic factors, or changes in genotype [1]. The analysis of metabolites can provide unique insights into biological processes since the metabolome represents the final readout of any biological system [2]. Especially untargeted metabolomics experiments generate thousands of features [3], which may be unknown at the time of measurement and may turn interesting in later studies. Unlike targeted liquid chromatography coupled to high resolution mass spectrometry (LC-HRMS (liquid chromatography coupled to high resolution mass spectrometry); a list of all abbreviations used throughout this manuscript is provided in “Abbreviations and Terminology”) approaches, an untargeted metabolomics experiment based on full-scan LC-HRMS also allows for retrospective data investigation. In order to get as much information as possible from a single experiment, it can be beneficial to compare novel biological information to former experiments, thus addressing novel biological aspects (e.g., newly identified metabolites) that have not been investigated in former experiments.

LC-HRMS is one of the most commonly employed analytical techniques in metabolomics research. Chemical compounds are separated by two orthogonal properties, i.e., retention time (Rt) and mass-to-charge ratio (*m*/*z*). Although modern LC-HRMS instruments are well established and robust, the generated raw data tend to show shifts and drifts in both the chromatographic as well as mass spectrometric dimension. The extent of these shifts is more distinct for samples which have been analyzed in different batches or when the time span of data acquisition was rather long (e.g., several weeks or months). Instabilities can be caused by: (i) the instrument (e.g., systematic mass shifts, column age and new hardware such as pumps); (ii) the chemistry of the sample and separation system; and (iii) random variations (e.g., temperature fluctuations) [4,5]. Furthermore, the ion species formed by the analytes can vary depending on the instrument’s condition (e.g., contaminations in the ion source) and the composition of the mobile phase or sample matrix. Apart from the sheer size of the data, which makes manual analysis impractical, the comparison of different experiments is aggravated by the aforementioned drifts and shifts.

Several algorithms and software tools have been developed to address this problem. Some of these algorithms are restricted to the use of the total ion current (TIC) chromatogram [6,7,8], whereas most of the more recent approaches do not limit themselves to the chromatographic dimension and also include information from the *m*/*z* dimension [9,10,11]. The process of reducing the aforementioned *m*/*z* and Rt shifts between different LC-HRMS chromatograms is called alignment. The majority of the available tools, such as XCMS [12], MZMine [13]/MZMine2 [14] or the MapAligner tool of OpenMS [15], use warping techniques [4]. Warping shifts, stretches and/or compresses one or both dimensions (i.e., Rt and *m*/*z*) simultaneously to improve data consistency across different datafiles under investigation [16]. The applied warping function can be constant (i.e., a constant shift value across the whole chromatogram), linear (i.e., a constant change in shift over time) or non-linear (i.e., the change in shift is not constant). While many advanced warping algorithms exist [9,17,18], most of them are refined versions of either correlation optimized warping (COW) or dynamic time warping (DTW). Existing simple solutions suffer from various shortcomings such as model assumptions that fail to capture the behavior of real-world data, e.g., when applying a linear Rt shift function. More sophisticated and complex algorithms can yield increasingly accurate results using the non-linear retention time correction function (e.g., by the use of polynomial functions or splines). However, they often have undesirably long runtimes [4], which, combined with the fact that many of them are solely command line-based and lack a convenient user interface, hinders them from being widely used in the broad field of metabolomics.

Here we present a software tool called MetMatch for the user-guided alignment of different LC-HRMS chromatograms. Chromatograms of interest can be aligned to a reference feature list consisting of reference metabolites and their ions (i.e., ID, *m*/*z*, retention time, etc.). The reference feature list can be generated manually or by processing single or multiple LC-HRMS chromatograms with one of the currently available software tools for untargeted metabolomics such as XCMS [12], MZMine2 [14], or MetExtract [19]. The alignment is performed using novel, efficient algorithms that correct offsets in the *m*/*z* as well as non-linear shifts in the Rt dimension. Apart from *m*/*z* and Rt shift correction, the formation of different ion species is also accounted for, which is a distinctive feature of MetMatch. Users can tweak all processing parameters through a simple, convenient and interactive user interface.

The software is available free of charge for academic, non-commercial use and can be downloaded for Windows from [20]. Here, the software is exemplified with LC-HRMS data from untargeted metabolomics experiments investigating phenylalanine-derived metabolites in wheat and T-2 toxin/HT-2 toxin detoxification products in barley as well as artificial test data.

## 2. Materials and Methods

### 2.1. Computational Workflow

**Figure 1:**
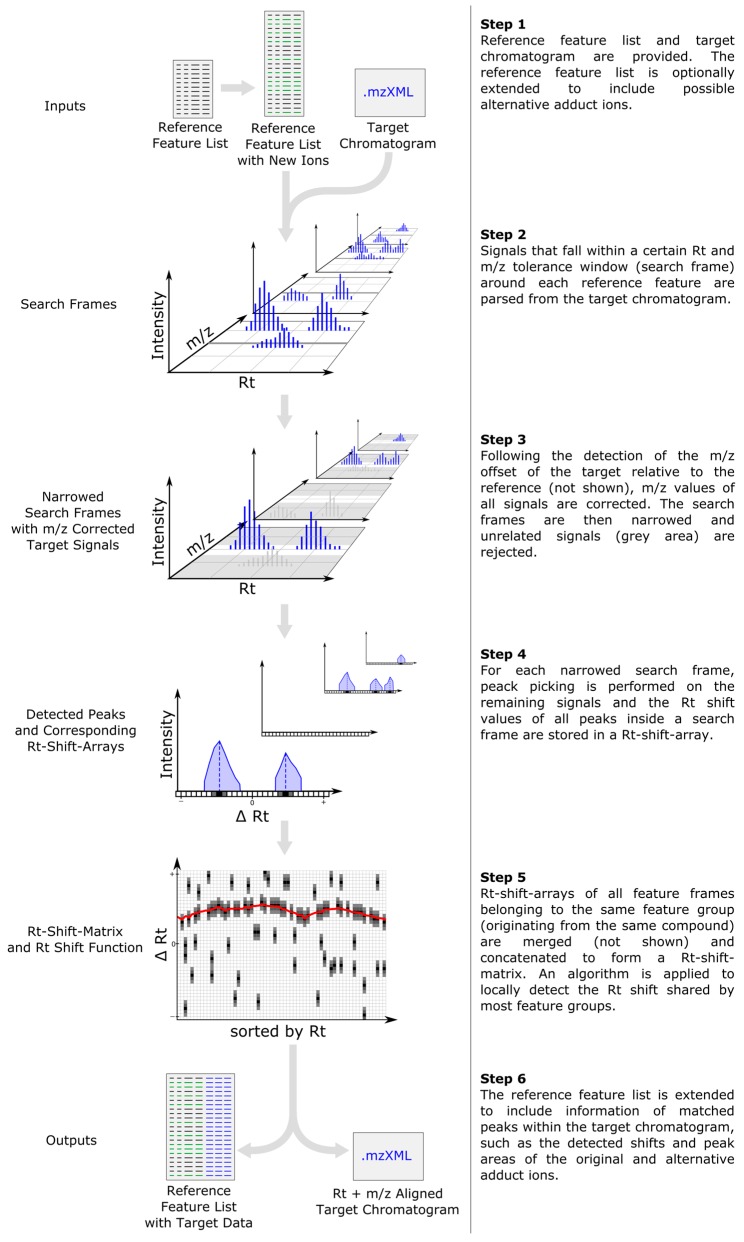
Workflow depicting the main data processing steps of MetMatch for a single target chromatogram. If more than one target chromatogram is going to be processed, each of those is successively carried through the steps 2–5 and a single data table containing the (extended) reference list as well as the matched features of every target chromatogram is generated. After processing of the target chromatograms, all new information (chromatographic peak area, corrected *m/z* value and retention time, calculated *m/z* and Rt shift) for each reference feature and processed target chromatogram are saved as an extended data matrix. Moreover, the target chromatograms can optionally be saved as *m/z*- and Rt-shift-corrected mzXML files.

The general data processing steps performed by MetMatch are outlined in Figure 1. Apart from generation and import of the reference feature list and output of the resulting data matrix, all processing steps are successively performed on a single datafile and then repeated for every file of a processed dataset. Details regarding the individual processing steps are provided in the following subsections.

### 2.2. Inclusion of Alternative Ion Forms (Step 1)

For each feature specified in the reference feature list, MetMatch generates an extended list of putative other ion adducts to account for differences in ion adduct formation between reference and target. These are typically caused by changes in eluent or other instrumental settings. Possible alternative ion species are provided by the user. If the ion species of a reference feature is known, MetMatch generates new ions based on the mass of the non-charged compound. In case the ion species of the reference feature is unknown, MetMatch first assumes this feature to be any ion species from the user-defined list, and then calculates the feature’s neutral mass and the *m*/*z* values of all putative ion species that can potentially occur for the particular compound.

### 2.3. m/z Offset Detection (Steps 2 and 3)

**Figure 2:**
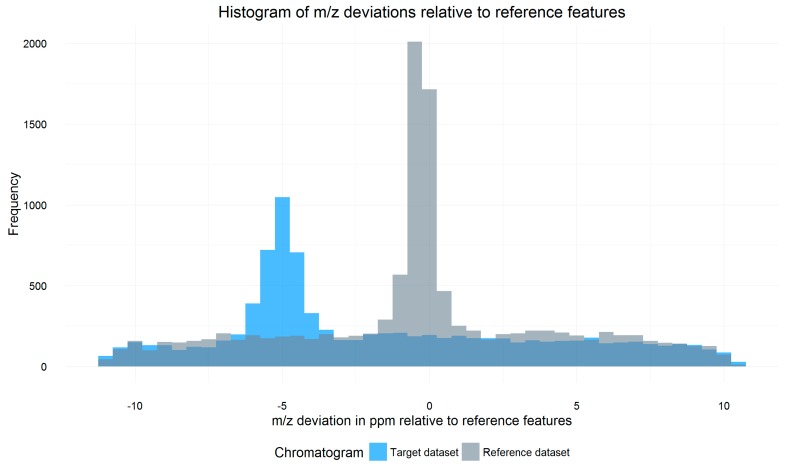
Overlay of two histograms showing the distribution of the weighted average mass to charge ratio (*m*/*z*) shifts of all search frames of a target file (blue) relative to a reference file (grey). The baseline frequency of about 200 is caused by unrelated signals, which have a random *m*/*z* shift relative to their respective reference feature. The *m*/*z* shift of signals corresponding to reference features is, however, not random and shows a significant increase around the systematic *m*/*z* offset of the respective file relative to the reference. This causes a maximum around the aforementioned offset. For the illustrated target datafile, this *m*/*z* offset was determined to be −5 ppm.

All signals of a target datafile that are within a certain Rt and *m*/*z* tolerance window (so-called search frame) around each reference feature are parsed from the respective datafile. For each search frame, the *m*/*z* shift of all included signals relative to the reference feature is calculated and weighted by the intensity of the signals. A histogram containing the weighted average *m*/*z* shift of all search frames of the datafile is then constructed. The *m*/*z* shift value of the bin with the highest frequency marks the constant systematic *m*/*z* offset of the respective datafile relative to the reference, as exemplified in Figure 2. While averaging *m*/*z* shifts between individual search frames helps to properly estimate the overall *m*/*z* offset, multiple chromatographic peaks with similar retention time and *m*/*z* values within single search frames or peaks partly split between neighboring search frames may cause conflicts which are not resolved by MetMatch or any other metabolomics data processing software.

After the detection of the *m*/*z* offset, *m*/*z* values of all signals inside the chromatogram are corrected by the calculated *m*/*z* shift. Corrected signals with an *m*/*z* deviation higher than a user defined ppm value are rejected, resulting in narrowed search frames.

### 2.4. Peak Picking (Step 4)

Following *m*/*z* offset detection, peak picking is performed on the remaining signals inside each narrowed search frame. Currently three different chromatographic peak picking algorithms have been implemented and can be selected in MetMatch. The MassSpecWavelet algorithm, while being very reliable, has significantly longer runtimes than the other two peak picking algorithms. These are based on Gaussian peak correlation and the Savitzky-Golay filter. For further details, please refer to Supplementary Materials Section 1.1.

### 2.5. Rt Shift Detection (Step 5)

**Figure 3:**
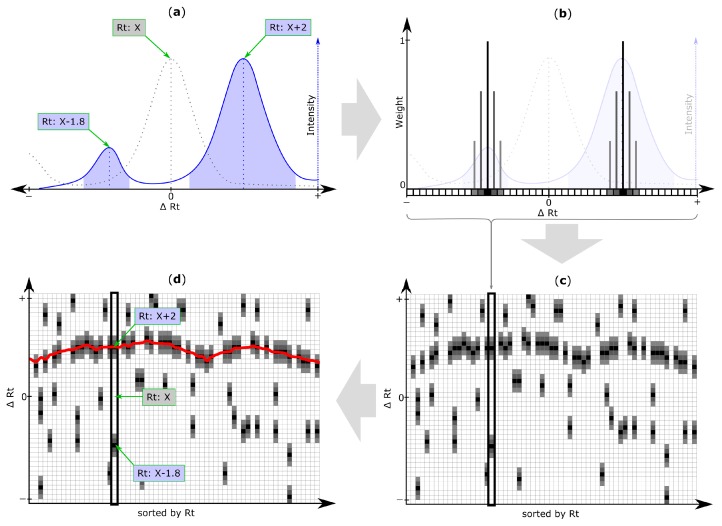
Retention time (Rt) shift detection procedure: (**a**) Extracted ion chromatogram (EIC) of a target chromatogram (blue solid line) with two chromatographic peaks (blue areas) and their peak apexes or centers (blue dotted line), depending on the selected peak picking algorithm. Determination of the chromatographic peak that correctly matches the reference peak (grey) is non-trivial, as the actual Rt shift is unknown; (**b**) All chromatographic peaks detected within a search frame are considered to be putative matching peaks and their Rt deviation relative to the original reference feature is discretized through binning. The resulting array of Rt shift bins is referred to as Rt shift array and is non-zero for all Rt shift bins at which—depending on the selected peak picking algorithm—the apex or center of the respective chromatographic peak has been detected. By default, the retention times of peaks are assigned a weight of 1 (black cells) and bins within a certain tolerance around the peak apex (or center respectively), are assigned linearly decreasing weight values (grey squares). All Rt shift arrays of search frames belonging to the same feature group (and therefore the same metabolite) are merged into a single array (step not shown); (**c**) All feature group Rt shift arrays are then sorted according to the Rt of their respective reference feature group and are concatenated column-wise to form the Rt shift matrix. Any non-zero entry in this matrix corresponds to the Rt shift (∆Rt) of a peak detected within a specific feature group (i.e., column) of the inspected target file. A region of higher density of putatively matching peaks marking the most probable Rt shift across the inspected chromatogram is clearly visible; (**d**) The Rt shift detection algorithm of MetMatch identifies this region of higher peak density by iteratively optimizing a function (red line) to be as close to as many peaks as possible, as outlined in Figure 4. This function is then used to determine the correctly aligned chromatographic peaks relative to their reference list entry. In the depicted case the aligned peak from the target chromatogram is X + 2.

Next, the Rt shift function is determined using all entries from the reference list. Figure 3a–c outlines the creation of the initial Rt shift matrix, containing Rt shift information of all peaks of a datafile detected during the peak picking step (step 4).

**Figure 4:**
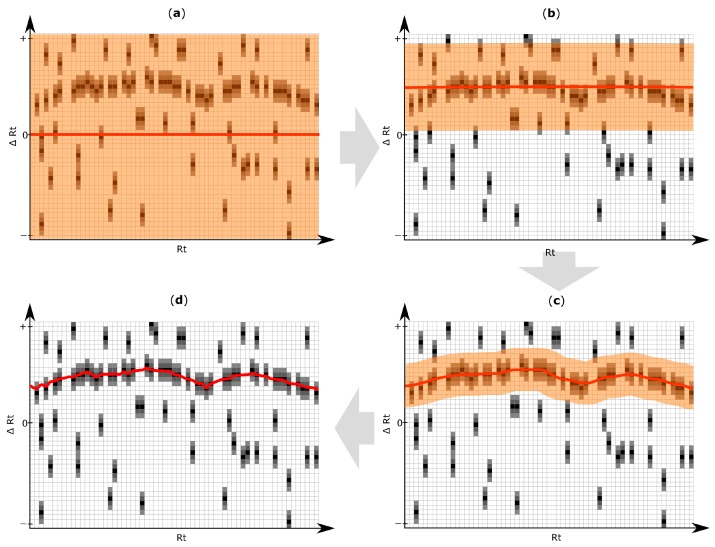
Exemplification of results obtained with the iterative retention time (Rt) shift detection algorithm. For improved illustration, only a certain part of the Rt shift matrix is depicted. (**a**) After construction of the Rt-shift matrix of the inspected target chromatogram (Figure 3a,b), the Rt shift function (red line) is initially constructed assuming no apparent shift (∆Rt = 0). The orange area highlights the maximum allowed Rt deviation which may be used in the next iteration step; (**b**–**d**) Three successive iteration steps were applied to model the most likely Rt shift of the target chromatogram relative to the reference feature list. The Rt shift matrix used in this exemplification consists of 45 rows and 63 columns. The three iterations of the Rt shift detection algorithm were using a row tolerance (orange area) of 23, 9 and 4 rows, and a column tolerance of 41, 4 and one column, respectively. Each iteration gradually adjusts the retention time correction function more to the region of higher peak density, which finally results in an optimal alignment maximizing the number of correctly aligned chromatographic peaks. The pseudocode of the algorithm for elucidation of the Rt shift correction function is listed in Supplementary Materials Figure S1.

An iterative algorithm is then applied to deduce an Rt shift function through the Rt shift matrix, as exemplified in Figure 4. The target peak that is closest to the Rt shift function but still within a user defined maximum tolerance is matched to the reference feature. For further details regarding the Rt shift detection algorithm, please refer to Supplementary Materials Section 1.2.

After several iterations of the chromatographic alignment algorithm, a sufficient agreement of the correction function with the region of high density of putative matching peaks has been achieved, which can also be visually inspected and accepted/rejected by the user (Figure 3d).

### 2.6. Output of Generated Results (Step 6)

After the *m/z* and chromatographic alignment steps have been determined, MetMatch applies these corrections to all chromatographic peaks from the reference feature list detected in the respective target chromatogram. This additional, new information (chromatographic peak area, corrected *m/z* value and retention time, calculated *m/z* and Rt shift) for each reference feature and processed target chromatogram are saved as an extended data matrix. Additionally, MetMatch also offers the option to save the processed target chromatograms as new mzXML files, in which all MS signals have been corrected by the determined *m/z* and Rt shifts.

### 2.7. Phe-Derived Biological Data

Two different test cases were chosen to demonstrate the MetMatch software tool. The first involves a dataset from a study investigating the metabolism of the aromatic amino acid phenylalanine (Phe) in wheat ears.

For generation of the reference feature list, wheat plants of the cultivar “Remus” were grown in a glass house under controlled conditions and inoculated with ^13^C_9_ phenylalanine solution (~99.5% ^13^C). Subsequent LC-HRMS measurement and TracExtract version 2 [21] data processing resulted in 151 Phe-derived features, which were detected in the ^13^C-Phe-treated sample. In order to increase the number of features on the reference list and thereby enhance *m*/*z* and Rt correction accuracy, plants of the same cultivar were also raised in a tailor made growth chamber with ^13^C enrichment of 98.8% as well as under native ^12^CO_2_ atmosphere in parallel. LC-HRMS analysis and data processing by AllExtract version 2 [22] resulted in 1482 features, which exclusively served to enlarge the reference list for matching Phe-derived metabolites in the samples of biological interest. The two resulting feature lists were merged (total of 1549 features) to create the reference feature list (for more details refer to Supplementary Materials Section 2.1) used in Section 3.8 as well as for artificial data evaluation (Section 3.10). The generation of the reference feature list is outlined in Supplementary Materials Section 2.2. It shall also be mentioned that only the information related to features of the native metabolite ions has been used by MetMatch.

For the target dataset, a study involving the two wheat cultivars “Remus” and “CM-82036” was chosen. Wheat plants (native, no ^13^C-labeling) were grown in a glass house, treated with water at the flowering stage and harvested 96 h after inoculation. These samples represent a part of an untargeted metabolomics experiment consisting of about 450 samples, which was carried out in the year 2012. Twenty of these samples have been used in the presented manuscript to demonstrate MetMatch. For more details on the experiment, refer to Warth et al. [23]. Samples were prepared and measured with LC-HRMS as described by Bueschl et al. [22] on an LTQ Orbitrap XL instrument in August and September 2012. Measurement of the two samples serving as the reference was carried out in December 2015. Between the measurement of the reference and target datasets, the pump of the respective HPLC had been replaced (Accela 600 pump to Accela 1250 pump) and the chromatographic column had been renewed (same type and dimensions, different production charge). All other method parameters and instrumental setup remained constant for all measurements.

### 2.8. T-2 Toxin/HT-2 Toxin-Derived Biological Data

The second test case chosen to demonstrate the developed tool is a study investigating plant-derived metabolites of the mycotoxins T-2 toxin and HT-2 toxin in barley reported by Meng-Reiterer et al. [24]. For comparison, matching of results and generation of the reference feature list, two barley samples were treated with native and ^13^C-labeled T-2 toxin and HT-2 toxin respectively. Measurements were performed using an LC-Q-TOF-system. The two chromatograms were processed using XCMS version 1.40.0, resulting in 20,682 features. T-2 toxin/HT-2 toxin-derived detoxification products reported by Meng-Reiterer et al. were matched to the list of barley metabolites. The time interval between generating the reference datafiles and measurement of the target files amounted to two years. Degradation of T-2 toxin/HT-2 toxin metabolites could therefore not be excluded. For further details regarding the generation of the reference feature list, please refer to Supplementary Materials Section 2.3.

## 3. Results and Discussion

We present MetMatch, a novel software tool for the comparison and alignment of LC-HRMS data from different metabolomics experiments. MetMatch is implemented in Java 8, as its performance is comparable to C/C++ while offering flexibility and abstraction from low-level operations [25]. Additionally, a plethora of high quality Java libraries exists, which helps to reduce development time. Java packages used by MetMatch are listed in Supplementary Materials Section 1.3.

**Figure 5:**
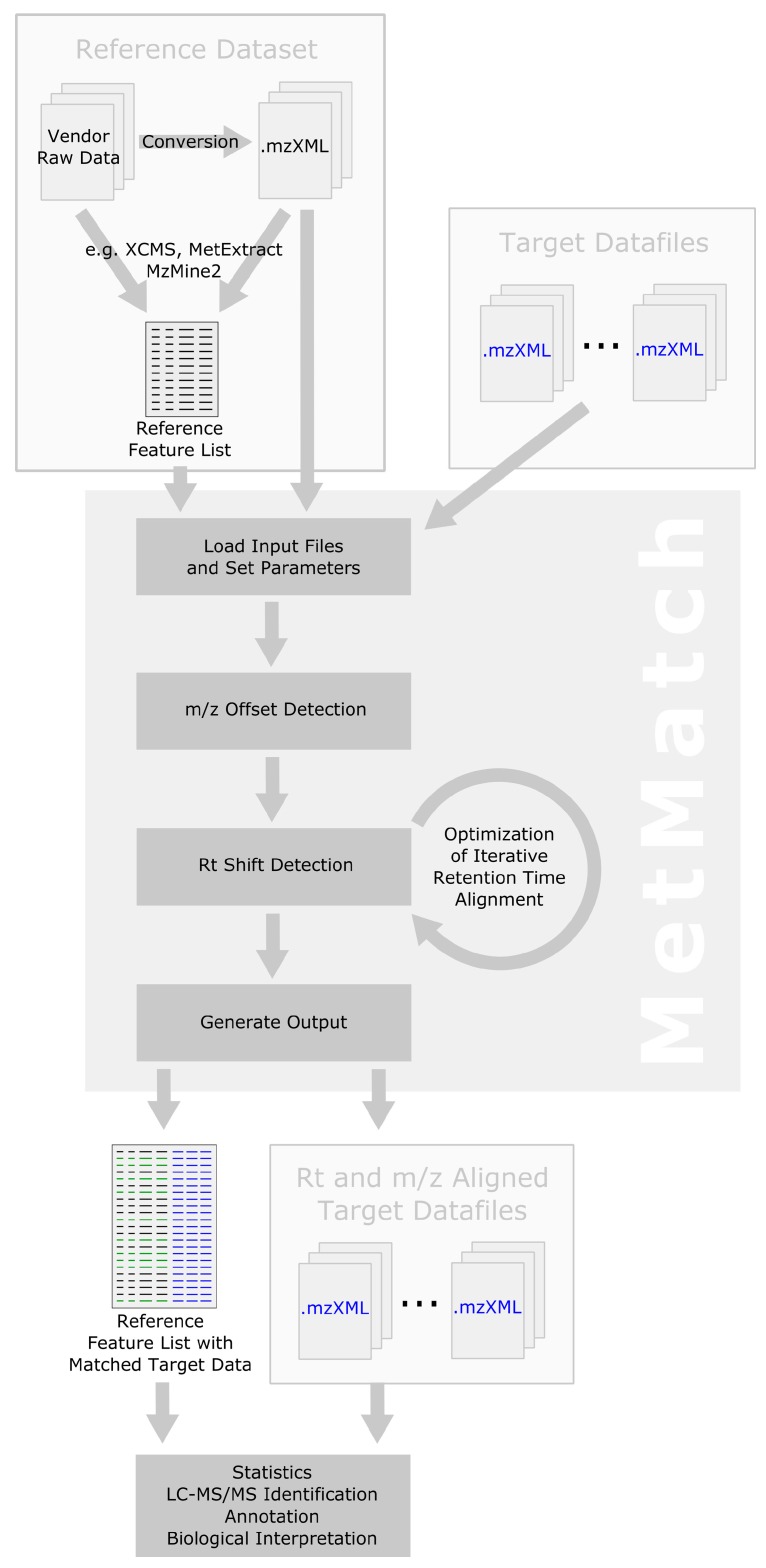
Schematic data processing workflow illustrating how MetMatch can be integrated into existing analysis pipelines.

Figure 5 shows a schematic representation of the MetMatch data processing workflow and how it can be integrated into already existing analysis pipelines. Refer to Section 2 for more details on the internal computational processing steps performed by MetMatch.

**Figure 6:**
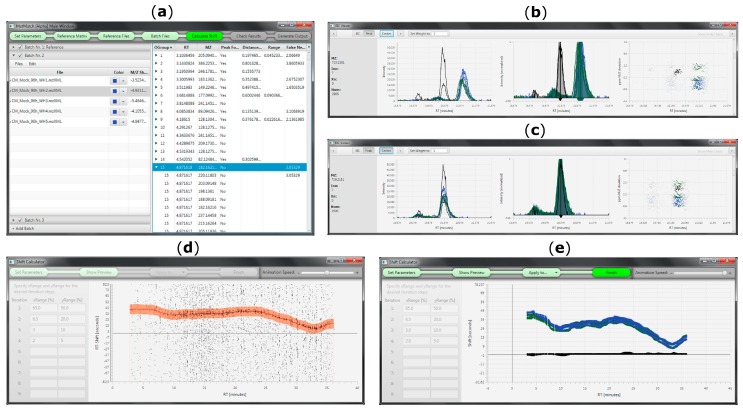
MetMatch’s user interface during various processing steps. (**a**) Main window showing the reference feature list and all loaded datafiles. The step indication bar on top helps the user to keep track of the data processing and also serves as a menu bar; (**b**) EICs of reference (black) and target datafiles (blue, green) before Rt alignment; (**c**) Overlay of EICs after Rt alignment; (**d**) Animated Rt shift detection algorithm (animated version available at [26]; (**e**) Overlay of Rt shift functions calculated for multiple target datafiles.

The purpose of the developed tool is to provide a fast, easy and accurate method to automatically detect *m*/*z* and Rt shifts between multiple measurements or LC-HRMS datasets, align all features between reference and individual target files and generate a table of matched metabolic features for further investigation (e.g., statistical processing, metabolite annotation). Due to the successive pairwise matching of target datafiles against a single reference list, MetMatch can also be applied to correct for drifts across a measurement sequence. This can be used to investigate if a dataset exhibited systematic changes in Rt and *m*/*z* shift over time. Usability is facilitated by a comprehensive and interactive user interface (Figure 6).

The main motivation behind MetMatch is to support the comparison of results between various metabolomics experiments, for instance the investigation of newly generated biological knowledge (e.g., novel or identified/annotated metabolites) in previous datasets or the comparison of LC-HRMS data from experiments with slightly modified biological conditions or sampling times. Section 3.8 and Section 3.9 demonstrate the flexibility of MetMatch with newly characterized, phenylalanine-derived metabolites as well as T-2 toxin/HT-2 toxin detoxification products in barley.

### 3.1. Accepted Input Files

MetMatch requires a list of reference features in the form of a tab-separated values (tsv) file. This list is typically obtained by processing one or more chromatograms with software for data processing of untargeted metabolomics approaches and serves as a reference for further metabolite matching in target datafiles. Each feature must be specified by the retention time (Rt), the mass-to-charge ratio (*m*/*z*), a unique identification number and the identification number of the associated feature group (Table 1). Because of the generic nature of the tsv file format, output from various applications such as XCMS [12], MzMine2 [14] or MetExtract [19] can be used as a reference either directly or after minor modification. Such a reference list can also be created from scratch using any text editor and should at least contain the information specified in Table 1.

The target chromatograms that are to be matched to the reference list have to be provided in the mzXML file format [27]. Most common vendor file formats can be easily converted to mzXML using freely available conversion tools such as ProteoWizard [28]. MetMatch also offers the option to load the unprocessed reference LC-HRMS chromatograms in the mzXML format for visualization. These are treated similar to target chromatograms by MetMatch and facilitate the visual assessment of EIC peaks underlying reference features as well as the success of the alignment process.

### 3.2. Detection of Differently Formed Ions

A chemical substance can form different ion species in the ion source of the mass spectrometer, such as protonated molecules ([M + H]^+^) or sodium ([M + Na]^+^) or ammonium ([M + NH_4_]^+^) adducts, each of which is detected at a unique *m*/*z* value. Especially when the ionization conditions vary between measurements (e.g., alteration of mobile phase composition or contamination of MS ion source), the relative fraction of the individual ion species may also change. Because of this, different ion species of the same metabolite might have been formed in the reference and target experiments, which poses a problem during comparison.

MetMatch addresses this problem by expanding the reference feature list with new alternative ion species. As a result, all ion forms specified on a user-created list are automatically searched for in the target files and matched against the extended reference feature list.

### 3.3. m/z Shift Detection

LC-HRMS data often exhibit small shifts in mass accuracy within analytical batches, even if the MS instrument has been calibrated immediately before the measurements. Therefore, *m*/*z* shifts have to be accounted for, especially when comparing different batches or longer sequences that do not use some method of online mass calibration. Shifts in the *m*/*z* dimension typically show negligible variation within a single file [4], which is why MetMatch assumes a constant *m*/*z* offset per file. This constant *m*/*z* offset is estimated using a histogram containing the weighted average *m*/*z* offset of all search frames of a chromatogram file. Search frames are defined as the set of all signals of a chromatogram that fall within a certain user-defined Rt and *m*/*z* tolerance around a reference feature. Using reference histograms, which are generated as outlined in Section 2.3, the *m*/*z* shift of the respective target file is determined, which is represented by the bin with the highest number of assigned ion signals.

### 3.4. Rt Shift Detection

While chromatograms within an analytical batch commonly only exhibit retention time shifts of small magnitude (usually less than the width of typical chromatographic peaks), this Rt shift can be more distinctive when comparing chromatograms from different analytical batches. In order to detect these Rt shifts, suitable alignment algorithms have to be employed [29]. MetMatch’s Rt shift correction method has been specifically developed for the detection of various forms of Rt shift, ranging from constant offsets to non-linear shifts in retention time.

It is assumed that most metabolites eluting within a narrow Rt range share a similar Rt shift between different chromatograms. If this assumption is wrong, every alignment attempt solely based on a single Rt shift function yields erroneous results [30]. Therefore, most of a chromatogram’s metabolite peaks should locally share an Rt shift, which can be described by a smooth function across a chromatogram. The Rt-alignment algorithm implemented in MetMatch uses an iterative approach to detect this Rt shift function. Since MetMatch provides an animated preview of the algorithm, the resulting function to be used for alignment of chromatograms can immediately be assessed visually and parameter settings can therefore be easily and quickly optimized by the user. When an appropriate balance between global and local alignment has been achieved, the user-guided settings may be applied to all samples in a dataset. The resulting alignment of each chromatogram can then be reviewed and adapted if necessary.

The applied Rt shift detection method has two limitations: (i) Depending on differences in their chemical structures, some metabolites may change their elution order due to variations in experimental conditions between the reference and target datafiles [30]. Based on Rt and *m*/*z* information alone, it is impossible to detect such changes, and therefore these features cannot be correctly matched; (ii) Reference list and target datafiles have to be compatible with regard to their features. If too few compounds/feature groups are shared between both of them, the quality of the resulting estimated Rt shift can decrease significantly. The minimum number of common feature groups has been exemplified with artificial data in Section 3.10.4. It is worth mentioning that not all features contained within the reference feature list have to be of biological interest. Additional features may be included only for the purpose of increasing the accuracy of the *m*/*z* and Rt shift detection. We have also chosen this approach in the two experiments described below to exemplify MetMatch.

### 3.5. Manual Corrections of Automatically Generated Results

All processing results can be verified and corrected manually using MetMatch’s interactive user interface. EICs are visualized and can be used to assess the initial signal extraction parameters as well as the quality of the detected peaks. The user is able to easily delete or manually pick peaks if necessary. Furthermore, the weight of individual EIC peaks can be increased according to their likelihood of representing the correctly matched metabolite ions. This way, the user can guide the automated Rt alignment algorithm with useful information in the form of user-verified “anchor” metabolites. Matched peaks are highlighted directly in the corresponding EIC’s and can be visually aligned to assess the automated processing result.

### 3.6. Output Format

The final result of MetMatch’s alignment routine is a tab-separated values (tsv) file containing the matched chromatographic peaks of all processed target chromatograms and datasets. It is essentially an extended reference feature list, containing the additional information derived from the matched chromatograms as exemplified in Table 2. If new ions were detected, they are included as additional rows in the data matrix. For each processed datafile, every matched chromatographic peak is specified by its EIC peak area, Rt and *m*/*z* value as well as their respective offsets by default. Additionally, *m*/*z*- and Rt-shift-corrected mzXML files can also be generated, which will contain all signals of the respective target chromatograms, however, their *m*/*z* and Rt values will be corrected using the MetMatch-derived shifts. Features which were not part of the reference list and were therefore not considered for the *m*/*z* and Rt correction by MetMatch are still corrected and linear interpolation is used whenever needed. These corrected mzXML files can then be viewed or further processed with other software tools capable of reading the mzXML format.

### 3.7. Runtimes

The runtime of MetMatch is linear with respect to the number of files and the number of entries in the reference feature list. Actual runtimes heavily depend on the applied peak-picking algorithm. For further details on the individual peak-picking algorithms, please refer to Supplementary Materials Section 1.1. Typical runtimes of the different algorithms for chromatographic peak picking on an Intel Core 2 Duo (2.8 GHz) are listed in Supplementary Materials Table S1.

### 3.8. Evaluation of the Phe-Derived Biological Metabolites

A metabolomics experiment studying phenylalanine (Phe)-derived metabolites in wheat was chosen as a reference. Seventy-five Phe-derived metabolites were detected and subsequently matched to a previous wheat experiment using MetMatch. Before matching, the reference list was extended to include metabolites that are not of interest (i.e., non-Phe-derived wheat metabolites), but help improve the accuracy of the *m*/*z* and Rt shift calculations. Refer to Supplementary Materials Section 2.2. for details on the creation of the reference feature list. While all features contained in the reference feature list have been used by MetMatch to correct the *m*/*z* and Rt shifts, data evaluation has been limited to the Phe-derived features since only those have been of scientific interest in the respective biological study. Rt shifts were to be expected since the two datasets were generated in the years 2012 and 2015. Furthermore, the HPLC pump and the chromatographic column had been replaced between the two experiments.

Processing all 22 files using MetMatch took 24 min on an Intel Core 2 Duo (2.8 GHz) with the parameters listed in Supplementary Materials Section 2.1. Matching chromatographic peaks for 92 of the 151 Phe-derived metabolite features contained in the reference feature list were detected in at least five target datafiles using MetMatch. Only 42 of these features were matched if differently formed ion adducts were not accounted for. On average, the software determined an *m*/*z* shift of −3.4 ppm as well as a non-linear retention time shift ranging from 7 to 52 s as illustrated in Figure S2 in the Supplementary Materials.

To compare the detected Rt shift of MetMatch with another commonly used software tool in the field of metabolomics, the same chromatograms have also been processed and aligned with XCMS. XCMS processing took 73 min on a similar computer (Intel Core 2 Duo, 2.9 GHz, no multithreading). However, XCMS uses an untargeted approach and therefore does not use the reference feature list. The resulting Rt shift functions obtained by MetMatch and XCMS agree across the majority of the Rt range of interest (3 to 36 min). Rt values smaller than 7 min, however, show reduced consensus. The underlying data shows only few signals in this Rt region, and it was not possible to definitively confirm or reject any of the two proposed Rt shifts by means of manual investigation. The calculated retention time shift functions of MetMatch and XCMS were compared using the Pearson correlation coefficient. For this, the calculated retention time shift functions of MetMatch and XCMS were compared pairwise. Between the retention time interval of 3 to 38 min, a mean correlation of 0.858 (standard deviation of 0.042) was calculated. However, for the retention time interval of 7 to 38 min, the average correlation increased to 0.926 (standard deviation of 0.011), which confirms that MetMatch better detected the retention time shifts in areas where more reference features are present and that the detected retention time shift functions of MetMatch and XCMS are very similar. A comparative illustration of the retention time shifts detected by MetMatch and XCMS is shown in Figure S2 in Supplementary Materials.

MetMatch’s capability of matching different ion species has been tested by means of matching the same target samples as before and additionally using chromatograms measured in negative ionization mode. The used reference feature list only contained ions measured in positive ionization mode. Matching them to the samples obtained in negative ionization mode requires the inclusion of alternative negative adduct ions in the reference feature list. This was automatically done by MetMatch as described in Section 2.2. Possible positive (e.g., [M + H]^+^, [M + Na]^+^, [M + NH_4_]^+^) as well as negative (e.g., [M − H]^−^, [M + Na − 2H]^−^, [M + Cl]^−^) ion species had to be provided by the user. Figure S3 in Supplementary Materials shows a comparison of the Rt shift functions obtained by matching positive and negative files to the positive reference feature list. The resulting Rt shifts for positive and negative chromatograms agree well. The Pearson correlation coefficient for chromatograms obtained in the positive and negative ionization modes for the same sample were calculated. On average, a correlation of 0.826 (standard deviation of 0.038) was calculated for the retention time interval of 3 to 38 min. The correlation improved to an average of 0.977 (standard deviation of 0.008) when the retention time interval of 7 to 38 min was used, in which most of the features of the reference feature list were present. This demonstrates that MetMatch was able to match target files and a reference feature list that share no common ion species by extending the reference feature list with alternative putative ion species. Without accounting for differently formed ion species, matching positive and negative ionization data would not be possible in that way.

To validate the results generated by MetMatch, selected Phe-derived features have been manually investigated in the raw data of reference and target files (Thermo Xcalibur Software, version 2.2). To this end, the correctness and plausibility of the proposed *m*/*z* and Rt shifts was examined for 23 features in 10 files, ranging from low to high *m*/*z*- as well as Rt values. The calculated shift of 19 chromatographic peaks could be confirmed in all files. One peak could not be correctly matched in any file, because it was detected at a very early Rt. Very few other peaks were detected around this Rt, leading to an inaccurate Rt shift function for this Rt region. Two peaks were not matched in some files since their retention time shifts were slightly above the maximum allowed Rt shift tolerance around the Rt shift function. This could have been solved by increasing the tolerance, at the risk of including false positives. One peak could not be reliably matched in all files because of the peak shape (shoulder peak). For a table containing the manually investigated features, please refer to Supplementary Materials Table S2.

**Figure 7:**
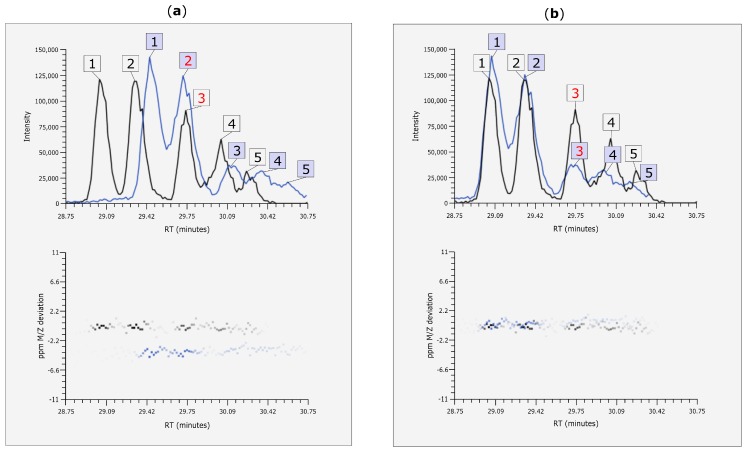
EIC (alignment before and after retention time (Rt) alignment and mass-to-charge ratio (*m*/*z*)) correction with MetMatch. (**a**) A phenylalanine-derived chromatographic peak was annotated at position 3 in the reference file (black) and thus included in the reference feature list. The target peak (blue) at position 2 would probably be matched to it by means of manual matching or any other alignment approach that does not account for Rt shift; (**b**) Extracted ion chromatograms (EICs) after Rt and *m*/*z* shift correction using MetMatch. When the two corrected EICs are viewed simultaneously, it is apparent that each target peak should be matched to the reference peak denoted by the same ID number and that a systematic *m*/*z* shift is present between the two chromatograms.

EICs of one of the manually validated chromatographic peaks are shown in Figure 7. Here, the usefulness of MetMatch becomes apparent, since manual comparison of the two experiments could have easily resulted in an incorrect match.

### 3.9. Evaluation of the T-2 Toxin/HT-2 Toxin-Derived Biological Data

In the second test case, 36 T-2 toxin/HT-2 toxin-derived metabolite ions were matched to a previous barley experiment (Supplementary Materials Section 2.3). Since two years have passed between the two experiments, possible Rt shifts were expected. The reference list was again extended to improve the accuracy of Rt correction. This time, all features detected with an untargeted approach facilitated by XCMS were used as the reference feature list. Processing all five files using MetMatch took 5 min on an Intel Core 2 Duo (2.8 GHz) using the parameters listed in Supplementary Materials Section 2.3. MetMatch determined a non-linear retention time shift ranging from −9 to −24 s as illustrated in Figure S4 in Supplementary Materials.

Again, the retention time alignment calculated by MetMatch was compared to that detected by XCMS for the same chromatograms. XCMS processing took 40 min on a similar computer (Intel Core 2 Duo, 2.9 GHz, no multithreading). However, it shall be mentioned that XCMS uses an untargeted approach and therefore does not make use of the reference feature list. The resulting Rt shift functions obtained by MetMatch and XCMS agree across the majority of the Rt range of interest (3 to 22 min). Rt values below 5 min, however, show no consensus. The underlying data show only few signals in this Rt region, and thus it was not possible to definitively confirm or reject any of the two proposed Rt shifts by means of manual investigation. Again, the Pearson correlation coefficient was used to calculate the similarity of the retention time shift functions calculated by MetMatch and XCMS. The average correlation was 0.966 (standard deviation of 0.013) in the retention time interval of 4 to 22.5 min, demonstrating that both MetMatch and XCMS calculated very similar retention time shift functions for the chromatograms. An illustration of the retention time shifts detected by MetMatch and XCMS is shown in Figure S4 in Supplementary Material.

Thirty-two out of 36 T-2 toxin/HT-2 toxin-derived metabolite ions were successfully matched in all target chromatograms. Three chromatographic peaks could not be correctly matched in any file because they were detected at an early Rt. Too few other peaks were detected around this Rt, leading to an inaccurate Rt shift function for this Rt region. One chromatographic peak was missed in two of the files during peak picking, because of its low abundance (peak height lower than 3000 counts) and its poor peak shape.

### 3.10. Evaluation of Artificial Data

While the evaluation of real-world data can provide important insight into the general quality of the resulting matches, knowledge of the true shifts is required in order to properly assess the results generated by MetMatch. To this end, several artificial datasets have been generated to test MetMatch’s performance. For each of these artificial tests, the reference list used in the Phe experiment (Section 2.7) has been modified by applying various *m*/*z* and Rt shifts. The original, unmodified reference chromatogram served as the target. These two datasets were then used as input for MetMatch and the generated results were compared to the artificially applied modifications. This approach was used to assess the number of true positives (Tp), false positives (Fp), false negatives (Fn) as well as precision, recall and F-measure. When matching the chromatogram directly to the unmodified reference feature list, results for a match without the need to correct for Rt or *m*/*z* shifts were obtained. This also served as a benchmark for further tests. The results of this match are displayed in Table 3.

From the 1482 features in the reference list (originating from two separate datafiles, for more details see Materials and Methods), 1446 were correctly matched, while 36 could not be matched. The reference feature list is based on two chromatograms (Section 2.7), including some metabolites that were only detected in the chromatogram that was not used as a target here. Therefore, the corresponding EIC peaks of the underlying metabolites were not detectable during peak picking. Moreover, although both MetExtract and MetMatch applied the MassSpecWavelet peak-picking algorithm, slightly different results were obtained. Since the algorithm is applied in the context of two different software tools, which have very similar EIC extraction approaches but do not share a common implementation, the output of the peak-picking algorithm can vary slightly. Even though these chromatographic peaks could not directly be matched to features on the reference list, their expected Rts were correctly interpolated.

#### 3.10.1. Formation of Different Ion Species

The automated search for differently formed ion species of the same metabolite is a distinct feature of MetMatch. To estimate the performance of the applied technique, the *m*/*z* values of all features in the reference list have been modified. The goal was to simulate a situation in which all metabolites contained in the reference list constituted the same type of ion species (A1), while for all of these metabolites a single different ion species (A2) was assumed for the target datafiles. Thus, the extended lists contained the *m*/*z* values of hypothetical adducts, which are not part of the original chromatogram. The inclusion of A1 and A2 in the target ion list, which is used to detect alternatively formed ion species, should allow MetMatch to detect all features regardless of their (modified) ion species. The results of this performance test are displayed in Table 4.

MetMatch successfully matched 1446 chromatographic peaks to features although they were simulated to be present as different ion species. The results are identical to the benchmark evaluation (see above), suggesting that MetMatch is able to match features regardless of their differing ion adducts.

#### 3.10.2. *m*/*z* Shift Detection

**Figure 8:**
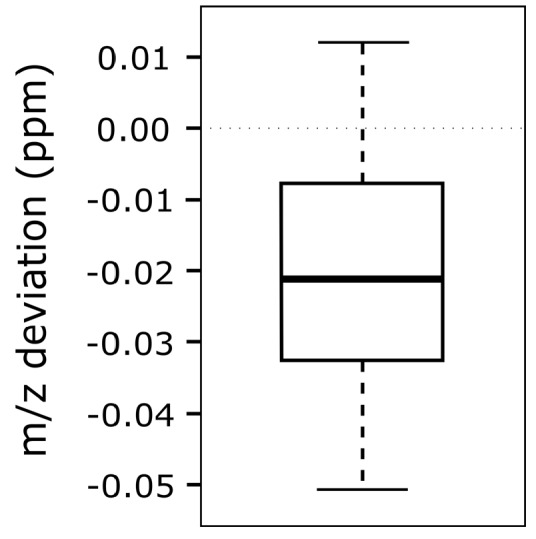
Box-plot showing the differences between imposed and detected mass-to-charge ratio (*m*/*z*) offset. A chromatogram was matched to 21 modified feature lists obtained by processing the same datafile with MetExtract and artificially imposing *m*/*z* offset values of −10 to +10 ppm, with 1 ppm increments per list.

To test the accuracy and reliability of *m*/*z* shift correction, various modified reference feature lists have been created which display *m*/*z* offsets of −10 to +10 ppm in 1 ppm incremental steps. In total, 21 different lists have been created to assess the difference between the artificially imposed *m*/*z* offset and the value calculated by MetMatch (Figure 8). The bins used in the underlying *m*/*z* offset calculation were set to a size of 0.01 ppm. Further decreasing the bin size did not increase the accuracy.

No correlation between the magnitude of artificially imposed *m*/*z* offset and the accuracy of MetMatch with respect to the correctly matched reference features in the target chromatogram has been observed. Differences between imposed and detected *m*/*z* offset were within the range of +0.01 to −0.05 ppm for all simulated cases. This is significantly less than the mass variation across a typical chromatographic peak recorded on the used LTQ Orbitrap instrument. In this test, the observed *m*/*z* discrepancy is explained by the fact that the *m*/*z* values of the reference features were calculated based on two chromatograms (Section 2.7). Thus, it is not surprising that the chromatogram, which was matched to this reference list, shows a marginal *m*/*z* deviation.

#### 3.10.3. Rt Shift Detection

**Figure 9:**
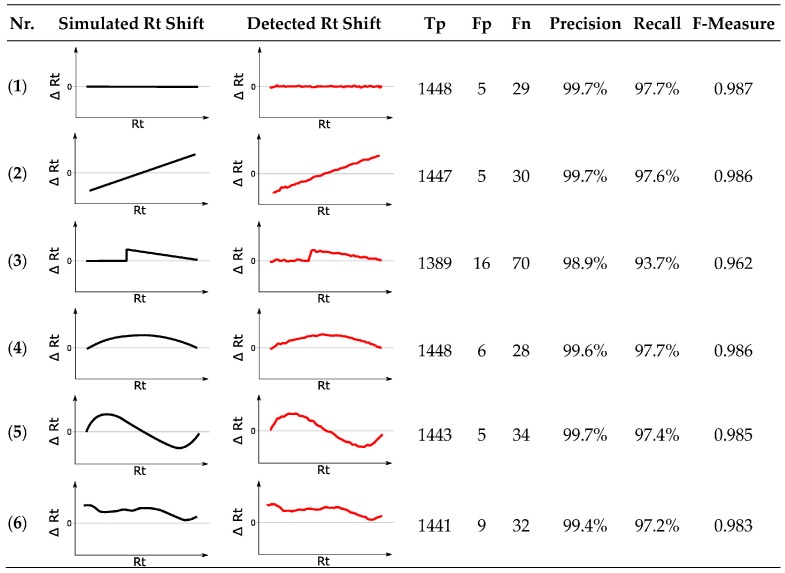
Results for six different simulated retention time (Rt) shifts. The reference chromatogram was matched to six modified feature lists, displaying artificially modified Rt shifts visualized by the black curves. The Rt shifts obtained by MetMatch are shown in red. (**1**) No Rt shift apart from random ± 6 s; (**2**) Monotonically increasing linear Rt shift; (**3**) Discontinuous Rt shift, as may, e.g., happen by a defective LC pump; (**4**) Non-linear Rt shift without inflection; (**5**) Non-linear Rt shift with inflection; (**6**) Complex non-linear Rt shift based on the type of Rt shift observed in the real-world wheat experiment.

Six modified reference feature lists were created displaying varying degrees of Rt shift relative to the original chromatogram. The applied retention time transformations are illustrated in Figure 9, column “Simulated Rt Shift”. On top of the respective systematic shifts, all feature groups were shifted randomly by ±6 s, simulating small Rt shifts observed in real-world data. Additionally, differently formed ion adducts were simulated analogous to Section 3.10.1.

Irrespective of the tested simulation, MetMatch achieved a high degree of correctly matched chromatographic peaks that are almost identical to the benchmark test. Occasionally, values exceeding the benchmark could also be observed due to slight variations in the generated EICs, which sometimes led to different peak-picking results. Although some features were incorrectly matched, the number of Fp (5 to 16) was quite low compared to Tp (1389 to 1448). Some of the Fp originated from the alternative ion species that were added to the reference feature list. In case of the modified reference feature list simulating a discontinuous Rt shift (simulation 3), the lowest number of correctly matched chromatographic peaks to reference features was observed. This is not surprising, since the algorithm tries to detect a continuous Rt shift (see Section 2.5) and only approximates the discontinuous shift applied in simulation 3. Especially around the erratic increase, MetMatch failed to fit the theoretical shift correctly. However, the number of 1389 correctly matched chromatographic peaks in contrast to 16 incorrectly matched peaks still corresponds to an F-measure value of 0.962. All other artificial Rt shifts resulted in F-measure values ranging from 0.983 to 0.987, which, compared to the benchmark F-measure value of 0.988, shows that MetMatch reliably detected the applied Rt shifts and produced high quality results.

#### 3.10.4. Minimum Required Common Feature Groups

**Figure 10:**
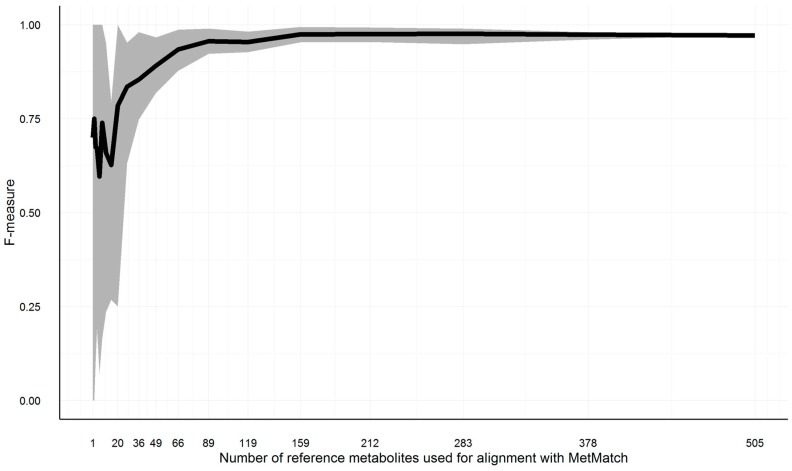
Illustration of the F-measure range (grey area) and average (black line) for reference feature lists of decreasing size. To estimate the minimum number of entries in the reference feature list, a simulation was carried out. In total, 200 subsets of decreasing size were generated. For each of the generated subsets, the *m*/*z* and Rt alignment step was calculated with MetMatch. The minimum (lower border of grey area), average (black line) and maximum (upper border of grey area) values are illustrated for each of the iterations.

As outlined in Section 3.4, a minimum number of feature groups has to be part of the reference feature list as well as the target chromatograms; otherwise, the Rt shift function cannot be reliably calculated. To test how large this number has to be in order to obtain reliable results, the reference feature list of Section 3.10.3, simulation 6, has been repeatedly matched to the original chromatogram, as described in Section 3.10. The number of common feature groups has been varied from 505 (complete list) to one. Starting from the complete list, subsets of decreasing size (each list being 25% smaller than its predecessor) have been created. This way, 20 reference feature lists with a decreasing number of common feature groups were simulated. Feature groups were deleted at random. This approach was repeated 10 times, cumulating in 200 MetMatch processing runs. The resulting F-measures for the various number of matched feature groups are shown in Figure 10.

On average, 159 common feature groups were required to maintain the same accuracy as achieved by 505 common feature groups. All test runs resulted in F-measures greater than 0.9 if at least 89 common feature groups were present. Decreasing the number to 49 common features groups still resulted in F-measures greater than 0.8 for all test runs. Below this number, the accuracy dropped more quickly. However, these numbers might vary for other datasets, as they are highly dependent on the number of isomers in each narrowed search frame (i.e., after *m*/*z* shift correction) as well as the number of narrowed search frames solely containing unrelated peaks. The higher these numbers, the higher the minimum required number of common feature groups.

## 4. Conclusions

Metabolomics is a valuable tool for studying phenotype changes [1] and is therefore widely used. Retrospective and comparative analysis of LC-HRMS chromatograms of separate biological experiments is of particular interest since they hold a considerable amount of information that can be used to investigate aspects beyond the scope of their initial acquisition and evaluation [2]. However, the reliable, comparative analysis is still an issue, due to shifts and drifts of retention time (Rt) and mass-to-charge ratio (*m*/*z*) or the formation of different ion species caused by the limited stability of experimental and instrument parameters. This holds true when comparing results obtained in multiple analytical batches using the same instrument, and especially when obtained by different instruments [5]. Several algorithms and software tools have been proposed to address this problem, but many of them suffer from severe drawbacks such as incorrect model assumptions and long run times [4].

In this respect, MetMatch is a novel software tool for the comparison of LC-HRMS data utilized through a convenient user interface. Chromatograms are matched to a feature list, which can be created manually or by processing one or multiple datafile(s), which serves as a reference. Constant shifts in *m*/*z* are automatically detected and corrected for. Both linear and non-linear Rt shift correction is achieved using a fast, iterative algorithm based on the observation that metabolites/compounds exhibiting similar Rt values within a measurement usually share analogous Rt shifts between measurements. Furthermore, MetMatch is able to account for the formation of different ion species that may be formed under slightly differing experimental conditions. The developed software offers a convenient graphical user interface that allows for easy optimization and verification of every processing step.

The quality of results obtained by MetMatch was assessed using two different test cases. The first case involved 75 newly characterized Phe-derived metabolites that were matched to a previous wheat experiment. Manual validation of 23 selected features was performed, 19 of which could be confirmed in all 10 inspected target files. Additionally, 36 T-2 toxin/HT-2 toxin-derived metabolite ions were matched to another barley experiment, 32 of which could be manually verified in all three target files. In both cases, MetMatch-derived Rt shifts were very similar to XCMS processing results. Furthermore, software evaluation by means of artificially manipulated reference data showed that MetMatch is well suited to account for the formation of different ion species between reference and target datasets and that both *m*/*z* and Rt shifts are detected with high accuracy (on average, 96.9% of all peaks were correctly matched).

In conclusion, we think that MetMatch will be a useful tool for the comparative analysis of metabolomics LC-HRMS experiments and we plan to add additional functionalities such as detection of non-linear *m*/*z* shifts and automatic assignment/correction of questionable matched features to further improve accuracy.

## Figures and Tables

**Table 1:** Structure of a typical reference feature list. Each feature is specified by four required values and four optional values relating to its ion species. “Ion ID” is an integer that uniquely identifies a feature. Each feature belongs to a feature group (i.e., metabolite), which is specified in the column “Group ID”. All features with the same “Group ID” are different ions of the same metabolite. “Rt” is the retention time of the feature in minutes and “*m*/*z*” is its mass-to-charge ratio. The remaining columns are optional and used for the generation of differently formed ions if specified.

Ion ID ^a^	Group ID ^a^	Rt ^a^	*m*/*z* ^a^	Ion Form ^b^	Uncharged Ion Mass ^b^	Ion Charge ^b^	Ionisation Mode ^b^
1	1	2.78	160.075	[M + H]^+^	159.068	1	+
2	1	2.78	177.102	[M + NH_4_]^+^	159.068	1	+
3	2	3.19	482.296	[M + NH_4_]^+^	464.262	1	+
4	3	3.27	330.458	[M + NH_4_]^+^	312.424	1	+

^a^ Mandatory information required by MetMatch; ^b^ optional information (used if provided)

**Table 2:** Detail of an output feature matrix generated by matching a target chromatogram to the reference feature list from Table 1. The columns “Ion Charge” and “Ionization Mode” have been omitted for simplicity. Features 901–903 are newly generated ion forms that were not contained in the reference list. Trying to align the target chromatogram to the reference without accounting for the formation of different ion species would have resulted in no matches for the displayed features.

Ion ID ^a^	Group ID ^a^	Rt ^a^	*m*/*z * ^a^	Ion Form ^a^	Uncharged Ion Mass ^a^	Reference Peak Area ^a^	File Peak Area ^b^	File Peak Rt ^b^	File Peak *m*/*z* ^b^
1 ^a^	1	2.78	160.075	[M + H]^+^	159.068	103,038	-	-	-
2 ^a^	1	2.78	177.102	[M + NH_4_]^+^	159.068	6,234,458	-	-	-
901 ^b^	1	2.78	182.057	[M + Na]^+^	159.068	-	3,423,472	2.83	182.057
3 ^a^	2	3.19	482.296	[M + NH_4_]^+^	464.262	523,238	-	-	-
902 ^b^	2	3.19	487.251	[M + Na]^+^	464.262	-	2,137,423	3.26	487.251
4 ^a^	3	3.27	330.458	[M + NH_4_]^+^	312.424	13,459,147	-	-	-
903 ^b^	3	3.27	487.251	[M + Na]^+^	312.424	-	15,358,291	3.38	487.251

^a^ Part of the reference feature list; ^b^ New column/row added by MetMatch.

**Table 3:** Benchmark results obtained by matching LC-full scan HRMS data of a wheat sample (serving as a target file) to a list of MetExtract-derived features from the same datafile (serving as the reference list).

Total Number of Features	Matched Features	Tp	Fp	Fn	Precision	Recall	F-Measure
1482	1446	1446	0	36	100%	97.6%	0.988

**Table 4:** Results obtained by matching LC full scan HRMS data of a wheat sample (serving as a target file) to a list of MetExtract-derived features from the same datafile (serving as the reference list) after modifying the *m*/*z* values to simulate the formation of completely different ion species in the reference and target datafile.

Total Number of Features	Matched Features	Tp	Fp	Fn	Precision	Recall	F-Measure
1482	1446	1446	0	36	100%	97.6%	0.988

## Supplementary Materials

The Supplementary materials are available online at http://www.mdpi.com/2218-1989/6/4/39/s1: Figure S1. Pseudocode of the iterative retention time (Rt) shift detection algorithm; Figure S2. Comparison of the Rt shift detected in the Phe-derived biological experiment using MetMatch and XCMS relative to the selected reference chromatogram; Figure S3. Comparison of the Rt shift detected in the Phe-derived biological experiment using chromatograms obtained in positive and negative ionization mode for the same samples; Figure S4. Comparison of the Rt shift detected in the T-2 toxin/HT-2 toxin–derived biological experiment using MetMatch and XCMS relative to the selected reference chromatograms; Table S1. Sample runtimes measured on an Intel Core 2 Duo (2.8 GHz); Table S2. Manual validation of the results obtained by matching the target dataset to the reference feature list containing Phe-derived metabolites.

## Abbreviations and Terminology

The following abbreviations and terminologies are used in this manuscript:

**Binning**	A range (e.g., *m*/*z* or Rt shift values) is divided into a series of equally long intervals (bins).
**Dataset**	A list of LC-HRMS datafiles belonging to one analytical batch, originating from the same experiment.
**EIC**	Extracted ion chromatogram. An EIC represents the abundance/intensity of a certain *m/z* value in each mass spectrum of a chromatogram. The mass spectra are sorted according to their acquisition time relative to injecting the sample into the HPLC instrument. Usually a small deviation from the selected *m/z* value (± ppm; depending on the used HRMS instrument) is allowed to account for minor scan-to-scan *m/z* inconsistencies observed during acquisition of a chromatogram.
**Feature**	A feature represents a particular metabolite ion of interest. It is characterized by its *m*/*z* value (experimental and theoretical) and retention time (as calculated by the used peak-picking algorithm). Features are usually the result of an untargeted metabolomics data processing approach and typically comprise multiple chromatographic peaks, one per LC-HRMS chromatogram.
**Feature group**	All features originating from the same chemical substance and hence sharing the same Rt and peak shape.
**F-measure**	Harmonic mean of precision and recall.
**Fn**	False negatives. Features that were not matched.
**Fp**	False positives. If a matched feature does not meet the criteria to be considered as Tp, it is an Fp.
**LC-HRMS**	Liquid chromatography coupled to high resolution mass spectrometry.
***m*/*z***	Mass-to-charge ratio.
**Peak/Chromatographic peak**	A chromatographic peak representing a particular feature in a chromatogram. Other than a feature, a peak has a defined *m*/*z* value, a retention time (as defined by the used peak-picking algorithm) as well as a peak area. Peaks are detected by the means of a peak-picking algorithm.
**Peak apex**	The time at which the most abundant signal of a respective chromatographic peak is detected after injection of the sample into the HPLC instrument.
**Peak center**	The time at which a chromatographic peak has its center of gravity. (Time) measurement is started with injecting the sample into the HPLC instrument.
**Precision**	Percentage of matched reference features that were correctly matched. Calculation: Number of Tp divided by the number of Tp plus Fp.
**Recall**	Percentage of all reference features that were correctly matched. Calculation: Number of Tp divided by the number of all reference features.
**Reference dataset**	A single or multiple LC-HRMS datafiles, which are used to generate the reference feature list.
**Reference feature list**	List of features to which target datasets are matched.
**Rt**	Retention time.
**Rt shift function**	A function that holds the calculated retention time shift between a target chromatogram relative to the reference feature list. It is used to map the retention times of detected chromatographic peaks in the target chromatogram to the retention time of the features in the reference list.
**Search frame**	Signals of a chromatogram (single datafile) that fall within a certain Rt and *m*/*z* tolerance window around a reference feature. These values have to be slightly larger than the maximum expected shift. (e.g., 1.5 min Rt shift and 10 parts per million (ppm) *m*/*z* shift for a typical LC-HRMS chromatogram).
**Rt shift array**	An array containing the Rt shift of all peaks detected within a search frame relative to the retention time of the respective reference feature. First, a search frame is discretized into smaller bins (number of bins is set by the user of MetMatch) each representing a certain Rt offset relative to the retention time of the respective reference feature. Then, those Rt bins for which chromatographic peaks of the respective reference feature were detected in the target chromatogram, are assigned weight values greater than zero (either automatically or user-guided). Any Rt offset bin for which no chromatographic peak was detected, is assigned zero value and thus do not contribute to the Rt shift detection algorithm. The number of entries in each Rt shift array is set by the user during the iterative Rt alignment step. Each entry in the Rt shift array corresponds to a certain Rt deviation between the retention times of the reference features and their corresponding chromatographic peaks in the target chromatograms.
**Rt shift matrix**	The individual Rt shift arrays of all reference feature groups are concatenated column-wise and sorted by the retention time of their reference feature group. Each column represents a feature group, while rows represent discretized Rt shift values.
**Target dataset**	A list of LC-HRMS datafiles to be matched to a reference feature list.
**Tp**	True positives. Matched features are considered Tp if their *m*/*z* deviation is ≤5 ppm and their Rt deviation is ≤8.4 s (manually validated threshold value for the distinction of Tp and Fp in this datafile) relative to the reference feature after shift correction. The formation of different ion species is taken into account when comparing the *m*/*z*.
