# Supplementary material for: MetMatch: A Semi-Automated Software Tool for the Comparison and Alignment of LC-HRMS Data from Different Metabolomics Experiments
Source: Metabolites. 2016 Nov 2;6(4):39. doi: 10.3390/metabo6040039 (PMC5192445; doi:10.3390/metabo6040039)
Supplement: Supplementary file 1 [file metabolites-06-00039-s001.pdf]

Supplementary Materials: MetMatch: A Semi-Automated Software Tool for the Comparison and Alignment of LC-HRMS Data from Different Metabolomics Experiments

Stefan Koch, Christoph Bueschl, Maria Doppler, Alexandra Simader, Jacqueline Meng-Reiterer, Marc Lemmens and Rainer Schuhmacher

1. Implementation Details of MetMatch

1.1. Peak-Picking Algorithms

Currently, three different chromatographic peak-picking algorithms have been implemented and can be selected in MetMatch. Table S1 shows an overview of the different algorithm's runtimes.

- MassSpecWavelet

  In order to provide a reliable peak-picking algorithm, Du et al. [1] implemented this continuous wavelet transform (CWT)-based algorithm that includes the consideration of the shape of the peaks. The retention time of a detected chromatographic peak is the peak's apex.

- Gauss Peak Correlation

  The extracted ion chromatogram (EIC) of a search frame is correlated to Gaussian peaks of different widths, after which the resulting array of Pearson correlations is smoothed using a moving average calculation. Minima and maxima of the correlation array are calculated using the first derivative and are then utilized to determine peak retention time and borders. The retention time of a detected chromatographic peak is the peak's center. This algorithm has been developed in-house.

- Savitzky-Golay Filter

  To further improve the performance of the above algorithm while keeping the general peak-picking principle, EICs are directly smoothed using a Savitzky-Golay filter [2]. Minima and maxima of the smoothed EIC are calculated using the first derivative and are then utilized to determine peak retention time and borders. The retention time of a detected chromatographic peak is the peak's center.

Table S1. Sample runtimes measured on an Intel Core 2 Duo (2.8 GHz). Reference Features: number of features in the reference feature list. Additional Adduct Ions: number of alternative adduct ions specified by the user. Total Features: number of features after alternative adduct ion generation. Note that the theoretical number would be higher, but some features have been rejected as their retention time and mass-to-charge ratio values are too similar to an already included feature. Runtimes for the individual peak-picking algorithms are displayed in hours:minutes:seconds.

Files	Reference Features	Additional Adduct Ions	Total Features	Runtime		
				MassSpecWavelet	Peak Correlation	Savitzky-Golay Filter
1	500	0	500	00:00:15	00:00:01	00:00:01
5	500	0	500	00:01:13	00:00:03	00:00:02
10	500	0	500	00:02:25	00:00:04	00:00:03
1	1000	0	1000	00:00:31	00:00:02	00:00:01
1	1500	0	1500	00:00:59	00:00:02	00:00:01
1	1500	3	9439	00:01:25	00:00:03	00:00:02
1	1500	5	28,660	00:02:38	00:00:04	00:00:02
1	20,000	5	394,110	01:56:13	00:08:10	00:01:28

1.2. Rt Shift Detection Algorithm

A novel, iteratively applied algorithm for the efficient calculation of the Rt shift present in a target chromatogram relative to the reference feature list has been developed. A matrix with peak shift information ($\boldsymbol{M}$), an array containing the current Rt shift function ($\boldsymbol{c}$) and two tolerance parameters ($\boldsymbol{x}$, $\boldsymbol{y}$) are required as inputs. The output is an array containing the updated Rt shift function ($\boldsymbol{c}'$). Figure S1 shows the pseudocode of the algorithm.

Input: $(\boldsymbol{M}, \boldsymbol{c}, x, y)$

matrix $\boldsymbol{M}$	*Rt-shift matrix. Columns represent the feature groups from the reference feature list and rows represent discretized Rt shifts relative to the retention time of the respective reference features. Each cell in the matrix with a value greater than 0 indicates a chromatographic peak in the respective target chromatogram that has a certain Rt-offset (=row-index) relative to its reference feature (=column-index). These values are weights, which have been set to 1 by MetMatch or higher values by the user.*
array $\boldsymbol{c}$	*current Rt shift values, corresponding to row indices in the matrix M, for each feature group of matrix M.*
integer x	*maximum allowed feature group tolerance, corresponding to column indices in the matrix M*
integer y	*maximum allowed Rt shift tolerance, corresponding to row indices in the matrix M*

start
    Initialize: array $\boldsymbol{weights}$, where $weights_f = 1$ for each $f \in \{1, \ldots, length[\boldsymbol{c}]\}$
    array $\boldsymbol{c}' =$ copy of $\boldsymbol{c}$

    for each $f \in \{1, \ldots, length[\boldsymbol{c}]\}$ **do**
        $maxproximity = 0$
        $newshift = -1$
        for each $r \in \{c_f - y, \ldots, c_f + y\}$ and r within borders of $\boldsymbol{M}$ **do**
(**a**) $proximity = \sum_{i=f-x}^{f+x} \sum_{j=c_f-y}^{c_f+y} \frac{M_{i,j}}{(|j-r|+1)^2}$ only for $M_{i,j}$ within borders of $\boldsymbol{M}$
            if $proximity > maxproximity$ **then**
                $maxproximity = proximity$
                $newshift = r$
            end if
        end for
        if $newshift > -1$ **then**
            for each $i \in \{f - x, \ldots, f + x\}$ and i within borders of $\boldsymbol{M}$ **do**
                $xdistance = \frac{x+1-|i-f|}{x+1}$
(**b**) $c'_i = \frac{c'_i * weights_i + newshift * maxproximity * xdistance}{weights_i + maxproximity * xdistance}$
                $weights_i = weights_i + maxproximity * xdistance$
            end for
        end if
    end for
end

Figure S1. Pseudocode of the iterative retention time (Rt) shift detection algorithm. An Rt shift matrix $\boldsymbol{M}$, an array $\boldsymbol{c}$ containing the current Rt shift for each column of the matrix and two range parameters $\boldsymbol{x}$ and $\boldsymbol{y}$ are required as inputs. The algorithm optimizes the current Rt shift $\boldsymbol{c}$ to be as close to as many chromatographic peaks as possible in the respective calculation window (a box drawn around each reference feature). The range parameters determine the size of this calculation window. The maximum Rt deviation from the current solution is set using the row range ($\boldsymbol{y}$). The column range ($\boldsymbol{x}$) allows optimizing for local maxima (low values) or more global maxima (high values). (**a**) Detection of the Rt shift value shared by most of the peaks within the calculation window is achieved by minimizing the squared distance to all peaks in the current window. (**b**) The final Rt shift of a feature group corresponds to the weighted average of the Rt shift of all feature groups

within the column range. The weight linearly decreases with distance, and increases proportional to the weight of all peaks in close proximity to the respective Rt shift.

1.2.1. Description of Input

Each column of the matrix $\boldsymbol{M}$ represents one feature group from the reference feature list, while each row of matrix $\boldsymbol{M}$ corresponds to a specific Rt shift value. A cell in matrix $\boldsymbol{M}$ holds the maximum weight of all peaks of a feature group (specified by its column) that have a certain Rt shift (specified by its row) relative to the reference. A value greater than 0 in a cell of the matrix $\boldsymbol{M}$ therefore indicates that a chromatographic peak for the respective metabolite has been detected in a certain target chromatogram at a certain retention time offset corresponding to the row index of the cell. Columns are sorted by Rt of their respective feature group.

For example: The maximum allowed Rt shift tolerance is equal to 50 s, and the number of Rt shift bins is equal to 100. The first row would now hold the weights of peaks that have an Rt shift of >49 to 50 s. The Rt shift range for the second row is then equal to >48 to 49 s and so on. The middle row of the matrix, in this example the 50th row, corresponds to no retention time shift and covers the Rt shift of >0 to 1 s. A chromatographic peak detected at an Rt-shift of 20 s relative to the retention time of its reference feature would therefore be saved in the 30th row (corresponding to an Rt shift of >20 to 21 s) of the matrix $\boldsymbol{M}$. The cell of the 30th row and the respective reference feature (column in $\boldsymbol{M}$) would have a value of 1 if the user has not change the weight for that reference feature.

An entry or "column" of array $\boldsymbol{c}$ corresponds to the same feature group as the respective column of matrix $\boldsymbol{M}$. Each entry contains the current value of the Rt shift function of the respective feature group. The value is specified by the index of the Rt shift bin (row of matrix $\boldsymbol{M}$), but does not need to be an integer (interpolation between rows of the matrix $\boldsymbol{M}$ is performed for $\boldsymbol{c}$).

For example: For the previously depicted example of a chromatographic peak detected at an Rt offset of 20 s relative to its reference feature, the value in $\boldsymbol{c}$ for this reference feature is likely close to 30, since the 30th row in the matrix M corresponds to a relative Rt shift of >20 to 21 s. However, the actual value depends on the search window and on the neighboring chromatographic peaks in that region of the target chromatogram.

The tolerance parameter x specifies the maximum distance (in column indices of matrix $\boldsymbol{M}$) two feature groups can have for them to still contribute to the updated Rt shift value (stored in $\boldsymbol{c}'$) of one another. It therefore determines whether to optimize for a global fit (bigger values) or a more local fit (smaller values).

The tolerance parameter y specifies the maximum distance from the current Rt shift value (in row indices of matrix $\boldsymbol{M}$ = Rt shift bins) a peak can have to still contribute to the updated Rt shift value of a feature group

1.2.2. Algorithm

Here, the algorithm presented in the pseudocode in Figure S1 is described in detail, referencing the current line in brackets. Generally, the algorithm updates the Rt shift function value c'_f of each feature group (for loop from lines **5** to **22**). The array $\boldsymbol{c}'$ is a copy of $\boldsymbol{c}$, and only written to but never read from an iteration of the algorithm. This way, intermediate results and the direction in which the matrix is processed do not influence the alignment iteration. Every c'_f is updated in the following way:

A box (i.e., calculation window) is considered around the current c_f whose dimensions are specified by the tolerance parameters x (first sum in line **9**) and y (second sum in line **9**). The Rt shift value $newshift$ that is in the center of the biggest cluster of peaks is detected (for the loop in lines **8** to **14**). This is done by iterating over all row indices r that are within the box. Next, the weight of all peaks within the box, divided by the squared row distance from the current r, is summed up and stored as $proximity$ (line **9**). Then, $newshift$ is assigned the row index r for which $proximity$ was maximized (line **12**). The distance is squared to promote the detection of clusters.

The $newshift$ of a feature group is not only used to update its own Rt shift function value c'_f, but also the value of neighboring feature groups (similar retention time shifts are assumed for local neighbors, for loop from lines **16** to **22**). Having found the Rt shift value $newshift$ that is within the biggest cluster of the box, the values of $\boldsymbol{c}'$ that correspond to any of the feature groups within the box are updated. The final value of c'_f is a weighted average of the $newshift$ values of all feature groups within the box. All contributing $newshift$ values are weighted by the column distance of their feature group to the current feature group ($xdistance$, line **17**) and the corresponding $proximity$. Rather than calculating the weighted average once the whole matrix $\boldsymbol{M}$ has been processed, calculation is performed using a running weighted average (line **19**). The current sum of weights for all c'_f is stored in the $\boldsymbol{weights}$ array (line **19**). The weight of each $newshift$ that contributes to the weighted moving average is calculated by multiplying the corresponding $proximity$ with $xdistance$ (lines **18**, **19**).

To summarize, a box (defined by the row and column tolerance) around the current Rt shift function value of each feature group is drawn. The biggest cluster of peaks inside this box is detected, and the Rt shift function values of all feature groups inside the box are updated.

1.3. Libraries and Tools Used in MetMatch

The following libraries and tools have been used in the presented implementation of MetMatch

- Apache Commons Math 3
  The normal distribution and Pearson correlation coefficient modules are utilized to calculate peak correlation with an idealized Gauss peak. Furthermore, linear interpolator is used to generate corrected mzXML files.
- JRI (Java/R Interface)
  The JRI package is used to interface MetMatch with an R-instance, as one algorithm for peak picking (MassSpecWavelet) is implemented in R.
- JavaFX
  MetMatch's user interface is set up with JavaFX, which is integrated into the Java Runtime Environment (JRE).
- ControlsFX
  This library is an add-on to JavaFX and includes many useful user interface control elements, resulting in a user-friendly and responsive interface.
- Java NIO
  The usage of memory-mapped files promises the possibility to store information on a disk (HDD/SSD) without discernible performance loss, as long as it is only processed occasionally.
- Michael Thomas Flanagan's Java Scientific Library
  Peak smoothing using either a moving average algorithm or a Savitzky-Golay filter is achieved through this library.
- uniVocity
  Input files are read and output files are formatted as tab-separated files (.tsv), which is performed with this efficient library.
- Maven
  The maven build system is used for managing project dependencies as well as building MetMatch.

2. Biological Experiments and MetMatch Data Processing

2.1. Phenylalanine-Derived Metabolites

2.1.1. Phenylalanine as Tracer

To provide insights into the set of Phe-derived metabolites of wheat, plants of the cultivar "Remus" were grown in a glass house under controlled conditions. At flowering stage which was reached after nine weeks, eight spikelets were inoculated with a 5g/l $^{13}C_9$ phenylalanine solution

(Cambridge Isotope Laboratories, ^{13}C enrichment of 99.5%). Seventy-two hours after inoculation, samples were harvested and immediately frozen in liquid nitrogen. For mock/control samples wheat ears were treated with water only.

2.1.2. Full Metabolome-Labeled Wheat Samples

^{13}C wheat plant ("Remus") was grown in a tailor made plant growing chamber (ECH-Halle, Halle, Germany) under controlled conditions (regulated temperature, light regime, nutrient solution, $^{13}CO_2$ concentration) for 62 days until the flowering stage (^{13}C enrichment of 98.8%). ^{12}C plants were grown under the same conditions.

The following *MetMatch processing parameters* were used to match the target dataset to the reference feature list:

Input Parameters

- Retention Time: RT
- Mass/Charge: MZ
- Ion ID: Num
- Metabolite ID: Group_ID
- Rest: (they were left blank)

General Parameters

- Start: 0
- End: 40
- RT Tolerance (Parsing Parameters): 1.5
- M/Z Tolerance (Parsing Parameters): 11
- M/Z Tolerance (Slice Parameters): 3.5
- Minimum Signals: 5
- Minimum Consecutive Signals: 3
- Intensity Cutoff: 5000
- Peak Picking: MassSpecWavelet
- S/N Threshold: 3
- Scales: 3, 19
- RT Tolerance (Rt Shift): 0.12
- Number of Bins: 100

Adducts

- Ion Name: 1M + H, Charge: 1+, Ion Mass: M + 1.007
- Ion Name: 1M + NH_4, Charge: 1+, Ion Mass: M + 18.034
- Ion Name: 1M + Na, Charge: 1+, Ion Mass: M + 22.989
- Ion Name: 1M + CH_3OH+H, Charge: 1+, Ion Mass: M + 33.033
- Ion Name: 1M +K, Charge: 1+, Ion Mass: M + 38.963
- Rest: (they were left blank)

Rt Shift Calculator Parameters

- 1st iteration: xRange: 65, yRange: 50
- 2nd iteration: xRange: 6.5, yRange: 20
- 3rd iteration: xRange: 3, yRange: 10
- 4th iteration: xRange: 2, yRange: 5

2.2. Creation of the Reference List (Phe Experiment)

2.2.1. Data Processing with TracExtract

Samples of wheat ears were searched for (^{13}C-) Phe-derived metabolites using an updated version of the in-house-developed TracExtract algorithm (unpublished software) [3,4].

- Search for Isotope Patterns from Native and ^{13}C-Labeled Metabolites

Signals of native and ^{13}C-labeled metabolite ions (M and M′) had to show a minimum abundance of 5000 counts, and a maximum *m*/*z* deviation of 1.5 ppm (intra-scan) between M and M′ was allowed with respect to the detected number of ^{13}C atoms. The isotopologs of both the native and labeled forms (M+1 and M′-1) were allowed to deviate up to 25% from their theoretical ratios for the detected number of ^{13}C atoms and no intensity threshold check was applied for these signals. TracExtract searched for six, seven, eight, and nine ^{13}C-atoms. Additionally, it also searched for 12, 14, 16, and 18 ^{13}C-atoms to be able to detect metabolites consisting of two ^{13}C-Phe units. Each such detected pair of signals (M and M′) was reported as MS signal pairs.

- Binning of MS Signal Pairs

Detected MS signal pairs were subsequently binned using hierarchical clustering (HC, Euclidean distance) using the *m*/*z* values of the native, monoisotopic isotopolog MS signals. After calculating the dendrogram, only those subclusters remained, which had less than 8 ppm *m*/*z* deviation between the MS signal pair with the highest and lowest *m*/*z* value.

- Detection of Chromatographic Peaks

Next, each subcluster from the previous binning step was used to calculate the EIC (±5 ppm) of the native, monoisotopic isotopologs as well as the EIC of the ^{13}C-labeled ions. The two EICs were then inspected separately for chromatographic peaks. Only those chromatographic peaks that were present in both EICs at approximately the same retention time (±10 scans) were considered to be derived from native and ^{13}C-labeled ions of the same metabolite. Next, the chromatographic peak shapes of such chromatographic peak pairs were compared using the Pearson correlation coefficient. Any putative peak pair not having a sufficient correlation (min. correlation: 0.85) was discarded, while those with a high correlation were reported as feature pairs of native and ^{13}C-labeled ion species and thus as ion species of Phe-derived metabolite ions.

- Convolution of Feature Pairs into Metabolite Groups

Detected feature pairs were then convoluted into feature groups again using the Pearson correlation coefficient. However, for this step not the chromatographic peak shapes of the native and the ^{13}C-labeled ions were compared, but rather the peak shapes of the chromatographic peaks of the native isotopologs of different feature pairs were compared. If their correlation was higher than 0.85, these feature pairs were said to be derived from the same metabolite and hence put into one feature group, each representing a single Phe-derived metabolite in the sample.

- Mock Samples Processing

To check for false positives the mock sample (water instead of ^{13}C-Phe) was processed in the same manner as the ^{13}C-Phe sample was processed.

- Generated Results

After data processing with TracExtract, a total of 151 feature pairs corresponding to 75 metabolites were detected in the ^{13}C-Phe sample as Phe-derived metabolites. In the mock sample, which was spiked with water instead of ^{13}C-Phe, eight feature pairs were detected. These eight feature pairs are false positives.

2.2.2. Data Processing with AllExtract

The AllExtract module of MetExtract was utilized to search for wheat metabolites using native and uniformly ^{13}C-labeled metabolite isotopologs [4,5]. Thus, any compound present in the LC-HRMS sample of the wheat ear was reported with this data processing strategy.

The search was performed as described earlier for the ^{13}C-labeled Phe-derived metabolites. However, as in these samples all metabolites were present as native and uniformly ^{13}C-labeled isotopologs, the number of carbon atoms was not restricted and AllExtract searched for a minimum of three and a maximum of 60 carbon atoms.

2.2.3. Combination of Phe-Derived Metabolites and Wheat-Metabolite Results

The 151 feature pairs detected with TracExtract (Phe-derived metabolites) and the 1482 feature pairs detected with AllExtract (all wheat metabolites) were merged into one reference feature list. The comparison of the two lists was done with a custom Python script, which compared the *m*/*z* values of the monoisotopic isotopologs as well as the retention time of putatively matched feature pairs. A maximum *m*/*z* deviation of ±5 ppm and a maximum retention time deviation of ±15 s were allowed for this matching.

2.3. T-2 toxin/HT-2 Toxin–Derived Metabolites

Reference and target datasets are based on an experiment performed by Meng-Reiterer et al. [6].

2.3.1. Reference Dataset:

The following reference files were used: one barley sample treated with native and ^{13}C-labeled T-2 toxin and one treated with native and ^{13}C-labeled HT-2 toxin. Both were measured on an LC-Q-TOF-system in positive full-scan mode with gradient method 2 and mass spectrometric settings according to Meng-Reiterer et al. [6]. All features present in the chromatogram were searched for with XCMS version 1.40.0 [7] and annotated with CAMERA version 1.20.0 [8]. Processing parameters were: peakwidth 10–45 s, ppm 5, prefilter c (5, 10,000), mzdiff 0.025, noise 2500, snthr 10, maxcharge 2. Using this approach, 20,682 features were detected and subsequently convoluted into 6764 metabolites. However, other than the TracExtract or AllExtract data evaluation strategies used for the Phe-derived metabolites, this list also includes different isotopologs and is therefore longer.

T-2 toxin/HT-2 toxin–derived detoxification products reported by Meng-Reiterer et al. [6] were matched to the list of barley metabolites using a maximum ppm deviation of five and a maximum retention time shift of 20 s. To match the peaks, the adducts $[M + H]^+$, $[M + NH4]^+$, $[M + Na]^+$ and $[M + K]^+$ were calculated.

2.3.2. Target Dataset:

The target dataset consisted of three measurements of one sample from the same dataset, which were performed on the same instrument two years later. Degradation of certain metabolites was therefore expected.

2.3.3. MetMatch Processing Parameters:

The following parameters were used to match the target dataset to the reference feature list:

Input Parameters

- Retention Time: rt_min
- Mass/Charge: mz
- Ion ID: Num
- Metabolite ID: pcgroup
- Rest: (they were left blank)

General Parameters

- Start: 0
- End: 25
- RT Tolerance (Parsing Parameters): 0.75

- M/Z Tolerance (Parsing Parameters): 70
- M/Z Tolerance (Slice Parameters): 40
- Minimum Signals: 5
- Minimum Consecutive Signals: 3
- Intensity Cutoff: 0
- Peak Picking: Savitzky-Golay Filter
- NU Threshold: 3
- RT Tolerance (Rt Shift): 0.14
- Number of Bins: 100

Adducts

- No additional adducts were generated

Rt Shift Calculator Parameters

- First iteration: xRange: 65, yRange: 50
- Second iteration: xRange: 6.5, yRange: 20
- Third iteration (only for reference files): xRange: 3, yRange: 10

3. Evaluation of MetMatch

3.1. Comparison of MetMatch with XCMS (Phe Dataset)

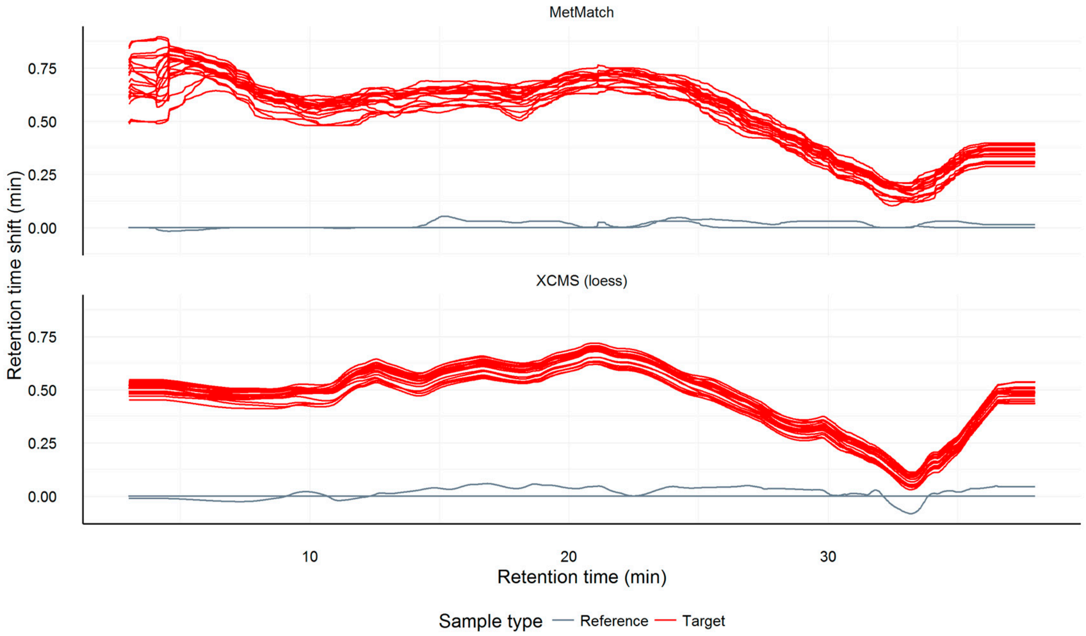

Figure S2. Comparison of the Rt shift detected in the Phe-derived biological experiment using MetMatch and XCMS relative to the selected reference chromatogram. Both tools calculated a similar Rt shift and thus a comparable chromatographic alignment for the two datasets relative to the selected reference chromatograms. The Pearson correlation coefficient was calculated pairwise for corresponding target chromatograms to compare the retention time shift functions calculated by MetMatch and XCMS. The correlation is on average 0.858 with a standard deviation of 0.042 for the retention time interval of 3 to 38 min. Between the interval of 7 to 38 min, which corresponds to the time interval in that most features of the reference feature list were detected, the average correlation for the target chromatograms was 0.926 with a standard deviation of 0.011. For the processing parameters of MetMatch see Supplementary Materials Section 2.1. Processing parameters for the XCMS peak picking were: findPeaks centwave, peakwidth 10–45 s, ppm 5, prefilter 5 × 10,000, noise 2500. Peaks before 3 and after 37.5 min were removed. XCMS group and Rt alignment parameters were: group density, bw 30, retcor loess, group density, bw 10.

3.2. Evaluation of Generation and Matching of Extended Reference Feature List

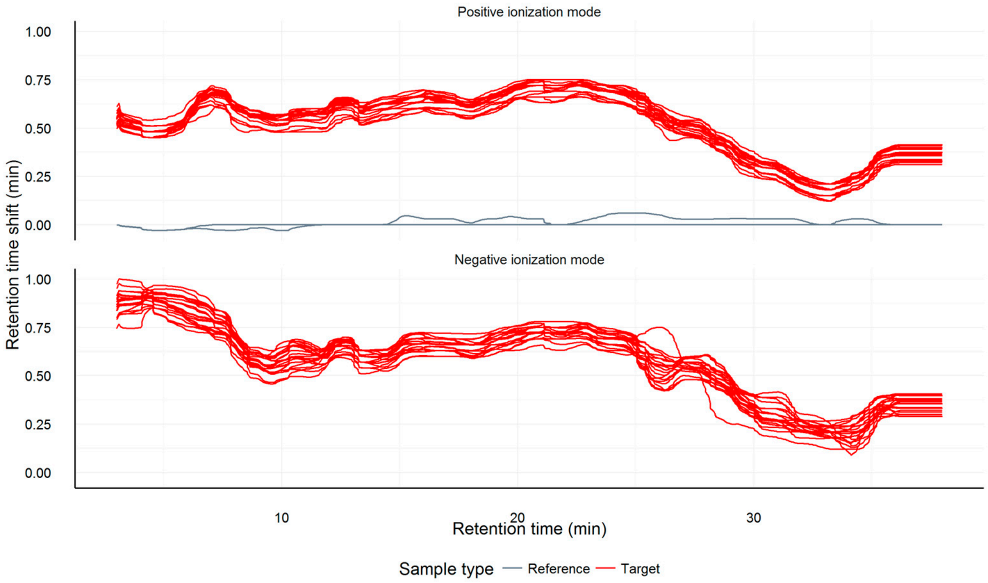

Figure S3. Comparison of the Rt shift detected in the Phe-derived biological experiment using chromatograms obtained in positive and negative ionization mode for the same samples. Reference files (grey) were obtained in positive ionization mode only. All target samples (red) of both ionization modes have been matched to the feature list exclusively containing ions detected in the positive ionization mode. The Rt shift functions of the negative and positive ionization mode chromatograms agree, except for retention times earlier than 7 min. This can be explained by the low number of reference features and chromatographic peaks in this region. One negative ionization mode chromatogram showed irregular behavior between Rt 15 to 35 min, which is explained by a reduced number of signals in this region of that particular chromatogram. The Pearson correlation coefficient was calculated pairwise for corresponding samples measured in the positive and negative ionization modes to compare the calculated retention time shifts. The correlation is on average 0.826 with a standard deviation of 0.038 for the retention time interval of 3 to 38 min. Between the interval of 7 to 38 min, which corresponds to that time interval in that most features of the reference feature list were detected, the average correlation for the target chromatograms was 0.977 with a standard deviation of 0.008.

3.3. Results of Manual Curation of Matched Phe-Derived Metabolites

Table S2. Manual validation of the results obtained by matching the target dataset to the reference feature list containing Phe-derived metabolites. Twenty-three matched features ranging from early to late Rt as well as low and high *m/z* values have been checked in 10 files (Remus, CM, 96h) using Thermo Xcalibur Software, version 2.2. Each feature is characterized by its unique identification number (Ion ID), the identification number its feature group (Group ID), its Rt and *m/z* values. Increasing the maximum allowed Rt shift tolerance would have resulted in successful matches of peaks 116 and 125 in all files. However, this could have caused an increased number of false positives.

Ion ID	Group ID	Rt	*m/z*	Correctly matched	Manual Validation Comment
23	19	5.48	105.0698	No	Peaks not found (too little features for such low *m/z* or Rt for correction function)
106	107	12.72	373.1129	Yes	-
108	107	12.72	390.1395	Yes	-
116	107	12.75	179.0701	Some	Not all peaks found because slightly out of maximum allowed Rt shift tolerance

125	107	12.74	197.0807	Some	Not all peaks found because slightly out of maximum allowed Rt shift tolerance
602	107	12.72	373.1128	Yes	-
1904	260	21.08	427.1384	Yes	-
1909	260	21.08	445.1492	Yes	-
1913	260	21.04	467.1309	Yes	-
1986	493	35.19	360.3258	Yes	-
2283	275	21.93	497.1441	Yes	-
2292	236	19.67	515.1544	Yes	-
3041	236	19.68	843.2318	Yes	-
3131	267	21.41	880.2867	Yes	-
3132	267	21.44	885.2420	Yes	-
10002	10002	4.15	120.0806	Some	Not all peaks found because it is a shoulder peak
10010	10010	7.78	207.1035	Yes	-
10011	10011	7.84	519.1971	Yes	-
10025	10025	13.23	274.0684	Yes	-
10030	10030	13.54	596.1689	Yes	-
10035	10035	16.50	640.1955	Yes	-
10059	10059	29.75	393.2609	Yes	-
10064	10064	34.88	834.5245	Yes	-

3.4. Comparison of MetMatch with XCMS (T-2 Toxin/HT-2 Toxin Dataset)

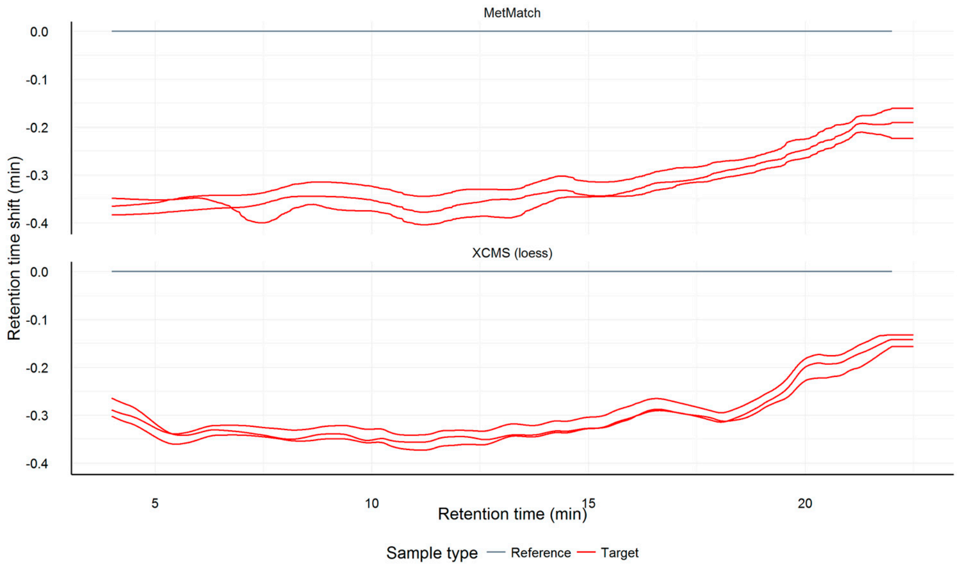

Figure S4. Comparison of the Rt shift detected in the T-2 toxin/HT-2 toxin–derived biological experiment using MetMatch and XCMS relative to the selected reference chromatograms. Both tools calculated a similar Rt shift and thus a comparable chromatographic alignment for the two datasets relative to the selected reference chromatograms. The Pearson correlation coefficient was calculated pairwise for corresponding target chromatograms to compare the retention time shift functions calculated by MetMatch and XCMS. The correlation is on average 0.966 with a standard deviation of 0.013 for the retention time interval of 5 to 22.5 min. For the processing parameters of MetMatch see Supplementary Materials Section 2.3. Processing parameters for the XCMS peak picking were: findPeaks centwave, peakwidth 10–45 s, ppm 5, prefilter 5 × 10,000, mzdiff 0.025, noise 2500. Peaks before 2 and after 22 min were removed. XCMS group and Rt alignment parameters were: group density, bw 30, retcor loess, group density, bw 10.

References

1. Du, P.; Kibbe, W.A.; Lin, S.M. Improved peak detection in mass spectrum by incorporating continuous wavelet transform-based pattern matching. *Bioinformatics* **2006**, *22*, 2059–2065.
2. Savitzky, A.; Golay, M.J.E. Smoothing and differentiation of data by simplified least squares procedures. *Anal. Chem.* **1964**, *36*, 1627–1639.
3. Kluger, B.; Bueschl, C.; Neumann, N.; Stuckler, R.; Doppler, M.; Chassy, A.W.; Waterhouse, A.L.; Rechthaler, J.; Kampleitner, N.; Thallinger, G.G.; et al. Untargeted profiling of tracer-derived metabolites using stable isotopic labeling and fast polarity-switching LC-ESI-HRMS. *Anal. Chem.* **2014**, *86*, 11533–11537.
4. Bueschl, C.; Kluger, B.; Berthiller, F.; Lirk, G.; Winkler, S.; Krska, R.; Schuhmacher, R. Metextract: A new software tool for the automated comprehensive extraction of metabolite-derived LC/MS signals in metabolomics research. *Bioinformatics* **2012**, *28*, 736–738.
5. Bueschl, C.; Kluger, B.; Lemmens, M.; Adam, G.; Wiesenberger, G.; Maschietto, V.; Marocco, A.; Strauss, J.; Bodi, S.; Thallinger, G.G.;et al. A novel stable isotope labelling assisted workflow for improved untargeted lc-hrms based metabolomics research. *Metabolomics* **2014**, *10*, 754–769.
6. Meng-Reiterer, J.; Varga, E.; Nathanail, A.V.; Bueschl, C.; Rechthaler, J.; McCormick, S.P.; Michlmayr, H.; Malachova, A.; Fruhmann, P.; Adam, G.; et al. Tracing the metabolism of ht-2 toxin and t-2 toxin in barley by isotope-assisted untargeted screening and quantitative lc-hrms analysis. *Anal. Bioanal. Chem.* **2015**, *407*, 8019–8033.
7. Smith, C.A.; Want, E.J.; O'Maille, G.; Abagyan, R.; Siuzdak, G. Xcms: Processing mass spectrometry data for metabolite profiling using nonlinear peak alignment, matching, and identification. *Anal. Chem.* **2006**, *78*, 779–787.
8. Kuhl, C.; Tautenhahn, R.; Bottcher, C.; Larson, T.R.; Neumann, S. Camera: An integrated strategy for compound spectra extraction and annotation of liquid chromatography/mass spectrometry data sets. *Anal. Chem.* **2012**, *84*, 283–289.

© 2016 by the authors. Submitted for possible open access publication under the terms and conditions of the Creative Commons Attribution (CC-BY) license (http://creativecommons.org/licenses/by/4.0/).
